# Epigenetic regulation of the nuclear-coded GCAT and SHMT2 genes confers human age-associated mitochondrial respiration defects

**DOI:** 10.1038/srep10434

**Published:** 2015-05-22

**Authors:** Osamu Hashizume, Sakiko Ohnishi, Takayuki Mito, Akinori Shimizu, Kaori Ishikawa, Kazuto Nakada, Manabu Soda, Hiroyuki Mano, Sumie Togayachi, Hiroyuki Miyoshi, Keisuke Okita, Jun-Ichi Hayashi

**Affiliations:** 1 Faculty of Life and Environmental Sciences, University of Tsukuba, 1-1-1 Tennodai, Tsukuba, Ibaraki 305-8572, Japan; 2 International Institute for Integrative Sleep Medicine (WPI-IIIS), University of Tsukuba, 1-1-1 Tennodai, Tsukuba, Ibaraki 305-8572, Japan; 3 Department of Cellular Signaling, Graduate School of Medicine, University of Tokyo, 7-3-1 Hongo, Bunkyo-ku, Tokyo 113-0033, Japan; 4 Subteam for Manipulation of Cell Fate, BioResource Center, RIKEN Tsukuba Institute, 3-1-1 Koyadai, Tsukuba, Ibaraki 305-0074, Japan; 5 Department of Physiology, Keio University School of Medicine, 35 Shinanomachi, Shinjuku-ku, Tokyo 160-8582, Japan; 6 Center for iPS Cell Research and Application, Kyoto University, Kawahara-cho 53, Shogoin, Sakyo-ku, Kyoto, 606-8507 Japan; 7 TARA Center, University of Tsukuba, 1-1-1 Tennodai, Tsukuba, Ibaraki 305-8572, Japan

## Abstract

Age-associated accumulation of somatic mutations in mitochondrial DNA (mtDNA) has been proposed to be responsible for the age-associated mitochondrial respiration defects found in elderly human subjects. We carried out reprogramming of human fibroblast lines derived from elderly subjects by generating their induced pluripotent stem cells (iPSCs), and examined another possibility, namely that these aging phenotypes are controlled not by mutations but by epigenetic regulation. Here, we show that reprogramming of elderly fibroblasts restores age-associated mitochondrial respiration defects, indicating that these aging phenotypes are reversible and are similar to differentiation phenotypes in that both are controlled by epigenetic regulation, not by mutations in either the nuclear or the mitochondrial genome. Microarray screening revealed that epigenetic downregulation of the nuclear-coded GCAT gene, which is involved in glycine production in mitochondria, is partly responsible for these aging phenotypes. Treatment of elderly fibroblasts with glycine effectively prevented the expression of these aging phenotypes.

The mitochondrial theory of aging proposes that age-associated overproduction of reactive oxygen species (ROS) and the resultant accumulation of somatic mutations in mtDNA are responsible for aging phenotypes including age-associated mitochondrial respiration defects^1^,^2^,^3^,^4^,^5^. This concept is supported partially by subsequent findings that mtDNA mutator mice expressing a proofreading-deficient mtDNA polymerase show accelerated accumulation of somatic mutations in mtDNA, resulting in the expression of mitochondrial respiration defects and premature aging phenotypes^6^,^7^,^8^. In contrasts, our previous studies proposed that the age-associated respiration defects found in human fibroblasts are caused not by mtDNA mutations^9^,^10^,^11^ but by nuclear-recessive mutations^11^. However, these findings can also be explained by assuming the involvement of epigenetic regulation of nuclear genes in the absence of nuclear-recessive mutations. Here, we addressed these controversial issues by reprogramming fibroblasts derived from elderly human subjects and examining whether age-associated mitochondrial respiration defects could be restored after the reprogramming.

## Results

### Characterization of human fibroblast lines

**Figure 1:**
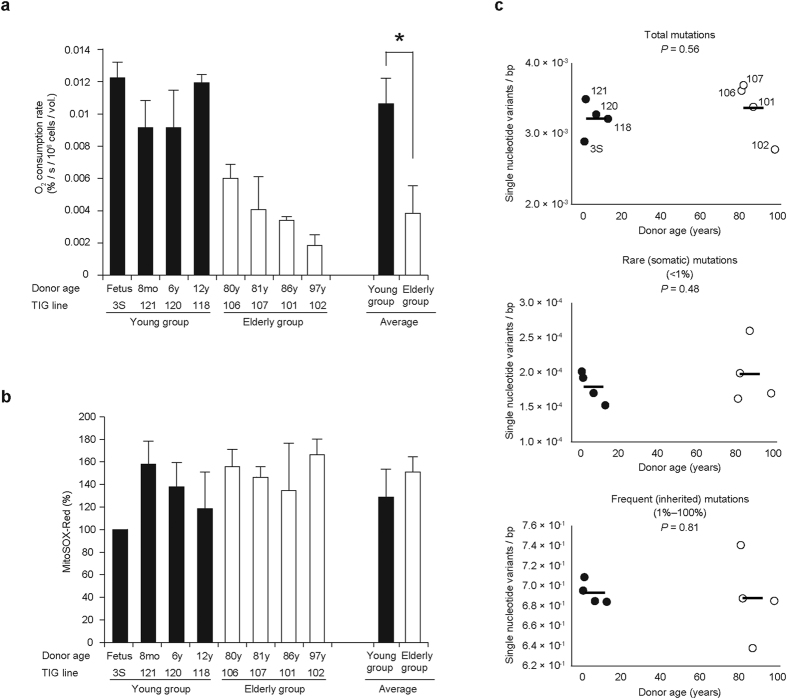
Examination of the mitochondrial theory of aging by using human fibroblast lines derived from young and elderly subjects. The ‘young’ group included fibroblast lines TIG3S (fetus), TIG121 (age 8 months [8mo]), TIG120 (6 years [6y]), and TIG118 (12 years [12y]). The ‘elderly’ group included fibroblast lines TIG106 (80 years [80y]), TIG107 (81 years [81y]), TIG101 (86 years [86y]), and TIG102 (97 years [97y]). (**a**) Comparison of mitochondrial respiratory function between young and elderly groups by estimation of O_2_ consumption rates. ‘Average’ indicates the average O_2_ consumption rates of each group. (**b**) Comparison of amounts of mitochondrial ROS (superoxide) between young and elderly groups by estimation of mitochondrial superoxide levels. Relative superoxide levels are expressed as mean fluorescence intensity of MitoSox-Red. ‘Average’ indicates the average fluorescence intensity of each group. Experiments in (**a**) and (**b**) were performed in triplicate; error bars, ± SD. *P < 0.05. Black and open bars represent young and elderly groups, respectively. (**c**) Comparison of mutation frequencies in mtDNA populations from young and elderly groups by using deep sequence analysis. Upper, middle, and lower panels represent frequencies of total, rare, and frequent mutations, respectively. Rare mutations existing in less than 1% mtDNA correspond to somatic mutations, whereas frequent mutations existing in 1% or more of mtDNA correspond to inherited mutations. Black and open circles represent young and elderly groups, respectively.

We used eight human fibroblast lines—four from young and four from elderly subjects—to examine the mitochondrial theory of aging. First, we examined mitochondrial respiratory function by estimating O_2_ consumption rates; we confirmed the presence of age-associated respiration defects in human fibroblast lines (Fig. 1a). However, the lines did not show age-associated increases in the production of superoxide (mitochondrial ROS) (Fig. 1b). Moreover, no decreases in mtDNA copy number (Supplementary Fig. 1a) or abnormalities in mitochondrial morphology (Supplementary Fig. 1b) were observed in the elderly fibroblasts.

We then quantitatively estimated the mutation frequency at each nucleotide position of mtDNA by deep sequence analysis of whole mtDNA prepared from the eight lines (Supplementary Fig. 2). The results unexpectedly showed that the total frequency of mtDNA mutations did not differ substantially between fibroblast lines from young and elderly subjects (Fig. 1c). Moreover, the frequency of rare mutations (existing in less than 1% of mtDNA), which have been proposed to be somatic mutations^12^, was not significantly greater in the fibroblast lines from elderly subjects than in those from young ones (Fig. 1c). The frequency of mutations existing in 1% or more of mtDNA, which have been proposed to be inherited mtDNA mutations^12^, also did not differ substantially between fibroblast lines from the young and elderly (Fig. 1c and Supplementary Table 1). Because we observed no age-associated overproduction of mitochondrial ROS and no age-associated accumulation of somatic mutations in the mtDNA of the human fibroblast lines we used (Fig. 1b and c), neither was associated with the mitochondrial respiration defects found in the fibroblast lines derived from the elderly subjects (Fig. 1a).

### Effects of redifferentiation of reprogrammed fibroblasts on respiration defects

Our previous report^11^ proposed that nuclear-recessive mutations are responsible for age-associated mitochondrial respiration defects in human fibroblast lines, because the respiration defects were restored by the introduction of pure nuclei (uncontaminated by mtDNA) from mtDNA-less HeLa cells into the fibroblasts. However, our previous results could also have been explained by epigenetic regulation of nuclear genes in the absence of nuclear-recessive mutations. In the case of epigenetic regulation, expression of mitochondrial respiration defects would be reversible and restorable with reprogramming.

**Figure 2:**
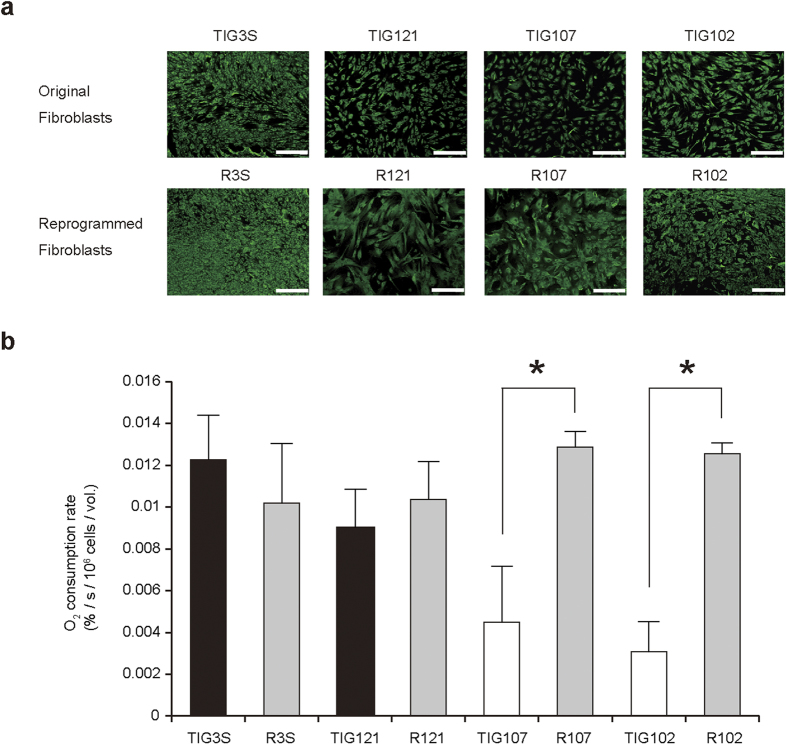
Effects of reprogramming of fibroblasts on age-associated mitochondrial respiration defects. (**a**) Immunostaining of original fibroblasts and fibroblasts redifferentiated from hiPSCs (reprogrammed fibroblasts) with antibody to a fibroblast-specific marker enzyme, namely the beta subunit of prolyl 4-hydroxylase. R3S, R121, R107, and R102 represent fibroblasts reprogrammed from the original fibroblasts TIG3S, TIG121, TIG107, and TIG102, respectively. Bars, 100 μm. (**b**) Estimation of O_2_ consumption rates of original and reprogrammed fibroblasts. Black and open bars are original fibroblasts from young and elderly subjects, respectively. Gray bars represent reprogrammed fibroblasts. Experiments were performed in triplicate; error bars indicate ± SD. *P < 0.05.

To examine this possibility, we randomly chose two young fibroblast lines (TIG3S and TIG121) and two elderly fibroblast lines (TIG107 and TIG102) and used them to generate human induced pluripotent stem cells (hiPSCs). These cells were then redifferentiated into fibroblasts and their mitochondrial respiratory function examined. For effective generation of hiPSCs from elderly human fibroblast lines, the conventional reprogramming gene set *OCT3/4*, *SOX2*, *KLF4*, and *C-MYC* required to isolate hiPSCs^13^ was replaced by the gene set *OCT3/4*, *SOX2*, *KLF4*, *L-MYC*, *LIN28*, and *p53shRNA*^14^. Moreover, virus vectors were replaced by episomal plasmids for transient introduction of the gene set into fibroblasts^14^. Small colonies with flat embryonic-stem-cell-like morphology (Supplementary Fig. 3a) were picked up about 4 weeks after transfection with the gene set. All the colonies expressed pluripotency marker genes of reprogrammed cells^15^, such as *Nanog*, *TRA-1-60* and *SSEA4* (Supplementary Fig. 3b), indicating that these colonies were hiPSCs. The cells were subsequently cultured in the absence of the feeder cells to allow their redifferentiation into fibroblasts^16^. The resultant growing cells were confirmed to be fibroblasts by their immunostaining with antibody to the beta subunit of prolyl 4-hydroxylase (Fig. 2a), which is a specific marker for fibroblasts^16^.

Next, we examined the O_2_ consumption rates of fibroblast lines redifferentiated from hiPSCs. The O_2_ consumption rates of redifferentiated fibroblast lines R107 and R102 were significantly greater than those in the original lines TIG107 and TIG102, respectively. Moreover, all of the fibroblast lines redifferentiated from hiPSCs had O_2_ consumption rates comparable to those of the fetal fibroblast line TIG3S, irrespective of whether they were derived from young or elderly subjects (Fig. 2b). Thus, the results in Fig. 2b reflect the reversibility of expression of age-associated mitochondrial respiration defects, indicating that these aging phenotypes are controlled by epigenetic regulation, not by mutations.

### Screening for nuclear genes regulating age-associated mitochondrial respiration defects

To identify nuclear-coded genes that were controlled epigenetically and could be responsible for age-associated mitochondrial respiration defects (Fig. 1a), we performed a microarray analysis and compared the gene expression spectra of the two young (TIG3S and TIG121) and two elderly (TIG107 and TIG102) fibroblast lines that had been used to isolate the hiPSCs (all microarray data were deposited at NCBI GEO database and received accession number GSE67000). Because our focus here was age-associated respiration defects, which are considered to be caused by a reduction in mitochondrial translation^10^,^11^, genes were selected by using the gene ontology (GO) term ‘mitochondria’ followed by four GO terms related to translation and respiration (Supplementary Fig. 4a). As a result, we selected 371 genes from among the 27,958 nuclear-coded genes used for the microarray analysis. From among these 371 genes, we furthermore selected six genes that showed age-associated regulation in the two sets of fibroblast lines with a log_2_ ratio of signal intensities of >0.585 or <–0.585, corresponding respectively to >1.5-fold upregulation or downregulation (Supplementary Fig. 4b).

**Figure 3:**
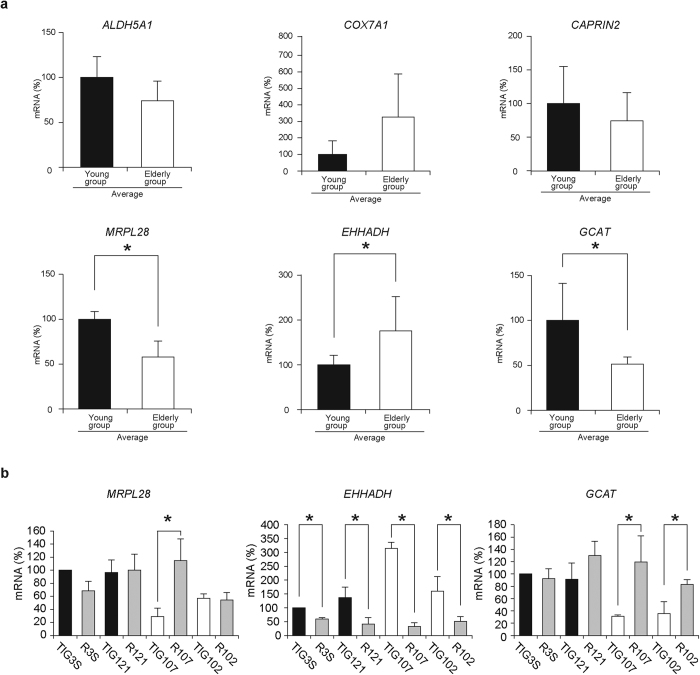
Use of real-time quantitative PCR to identify nuclear-coded genes regulating age-associated mitochondrial respiration defects. (**a**) Comparison of mRNA levels of the candidate six genes in the young and elderly groups. The six gene candidates for regulation of age-associated respiration defects were selected by using gene ontology terms and a microarray heatmap (Supplementary Fig. 4b). Black and open bars are the average gene expression levels of fibroblasts from young and elderly groups, respectively. The young group consisted of fibroblast lines TIG3S (fetus), TIG121 (age 8 months), TIG120 (6 years), and TIG118 (12 years). The elderly group consisted of lines TIG106 (80 years), TIG107 (81 years), TIG101 (86 years), and TIG102 (97 years). Levels of transcripts were normalized against *UBC* expression. The results of each fibroblast line were shown in the Supplemental Figure 5. Of the six genes examined, age-associate regulation was confirmed to be present in *MRPL28*, *EHHADH*, and *GCAT*. (**b**) Comparison of mRNA levels of *MRPL28*, *EHHADH*, and *GCAT* in original and reprogrammed fibroblasts. R3S, R121, R107, and R102 represent fibroblasts reprogrammed from the original fibroblasts TIG3S, TIG121, TIG107, and TIG102, respectively. Levels of transcripts were normalized against *UBC* expression. Black and open bars are fibroblasts from young and elderly subjects, respectively. Gray bars represent reprogrammed fibroblasts. Experiments were performed in triplicate; error bars indicate ± SD. *P < 0.05.

To confirm the microarray results, we performed real-time quantitative PCR to estimate the mRNA levels of the six genes. Comparison of their mRNA levels between the four lines from young subjects and the four lines from elderly subjects showed that *MRPL28* and *GCAT* were downregulated, whereas *EHHADH* was upregulated, in elderly fibroblast lines (Fig. 3a, Supplementary Fig. 5). In contrast, the remaining three genes showed no significant differences in age-associated regulation.

Because age-associated respiration defects were restored after reprogramming (Fig. 2b), we expected that the reprogramming of elderly fibroblasts would result in the reprogramming of age-associated down- or upregulation of the three genes. We examined this possibility by using redifferentiated fibroblasts from hiPSCs. Reprogramming of gene expression in elderly fibroblasts occurred in *GCAT* (Fig. 3b), which regulates glycine production in mitochondria^17^,^18^. It was therefore likely that reduced glycine production in mitochondria by epigenetic downregulation of *GCAT* (Fig. 3a) resulted in the age-associated respiration defects (Fig. 1a).

### Effects of down- or upregulation of the genes regulating age-associated respiration defects

**Figure 4:**
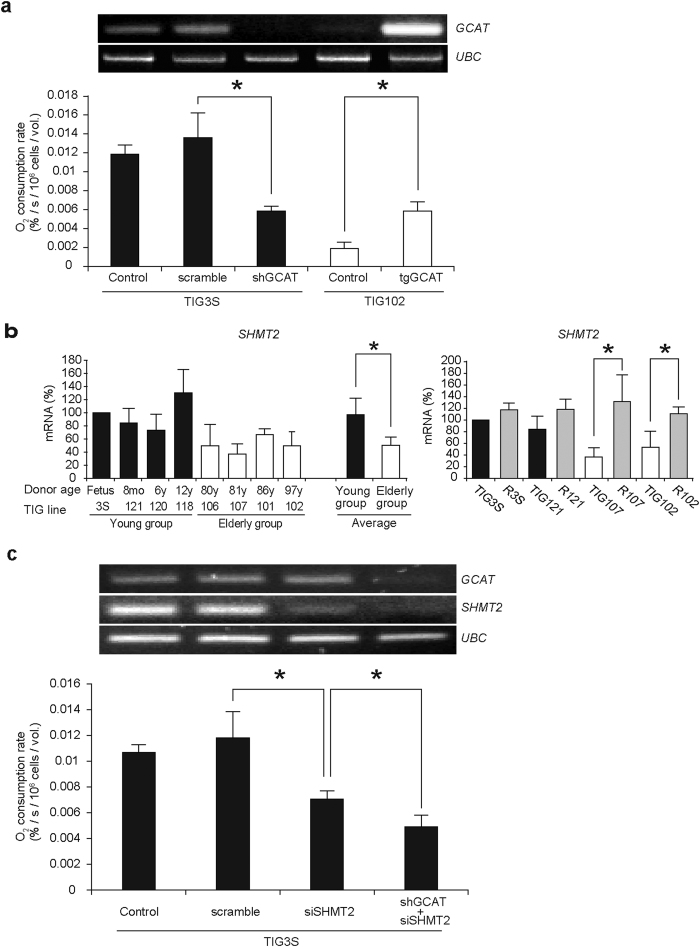
Effects on respiratory function of down- or upregulation of the genes regulating age-associated respiration defects. (**a**) Down- and upregulation of *GCAT* and their effects on respiratory function. Control, untreated; scramble, scrambled shRNA treated; shGCAT, downregulation of *GCAT* in fibroblast line TIG3S by using shRNA; tgGCAT, upregulation of *GCAT* in line TIG102 by using its cDNA-based transgene. Upper panel, mRNA levels; lower panel, O_2_ consumption rates. Black and open bars represent TIG3S and TIG102, respectively. (**b**) Comparison of mRNA levels of *SHMT2* in young and elderly groups (left panel) and in original and reprogrammed fibroblasts (right panel). Black and open bars are young and elderly groups, respectively. ‘Average’ in the left panel indicates the average gene expression levels of each group. R3S, R121, R107 and R102 (gray bars) in the right panel represent fibroblasts reprogrammed from TIG3S, TIG121, TIG107, and TIG102, respectively. (**c**) Downregulation of *SHMT2* or both *GCAT* and *SHMT2*, and their effects on respiratory function in TIG3S. Control, untreated; scramble, scrambled-siRNA-treated; siSHMT2, downregulation of *SHMT2* by siRNA; shGCAT + siSHMT2, simultaneous downregulation of *GCAT* and *SHMT2* by shRNA and siRNA, respectively. Upper panel, mRNA levels; lower panel, O_2_ consumption rates. Black bars represent TIG3S. Experiments were performed in triplicate; error bars indicate ± SD. *P < 0.05.

We then examined whether downregulation of *GCAT* in TIG3S (from a fetus) would induce respiration defects, and whether the gene’s upregulation in TIG102 (from an elderly subject) would restore reduced mitochondrial respiratory function. Downregulation of *GCAT* in TIG3S by using shRNA led to a reduction in mitochondrial respiratory function (Fig. 4a). Moreover, overexpression of *GCAT* in TIG102 by infection with lentivirus including the cDNA of *GCAT* restored the respiration defects (Fig. 4a). These observations suggest that epigenetic downregulation of *GCAT* with aging is responsible, at least in part, for age-associated respiration defects.

Because glycine production in mitochondria is regulated by *SHMT2*^17^ as well as by *GCAT*^18^, downregulation of *SHMT2* with aging may also be involved in age-associated respiration defects. To examine this possibility we used real-time quantitative PCR and compared the mRNA levels of *SHMT2* in the eight lines from young and elderly subjects. We found age-associated downregulation of *SHMT2* (Fig. 4b), even though our microarray results had not revealed its age-associated downregulation (Supplementary Fig. 3b). Moreover, reprogramming of fibroblast lines from aged subjects restored the reduced expression of *SHMT2* (Fig. 4b). Furthermore, downregulation of *SHMT2* in the TIG3S fibroblast line by using siRNA resulted in a reduction in respiratory function (Fig. 4c), and simultaneous downregulation of *GCAT* by using shRNA and of *SHMT2* by using siRNA had a synergic effect in reducing mitochondrial respiratory function (Fig. 4c). These observations indicated that epigenetic downregulation of both *GCAT* and *SHMT2* with aging (Fig. 3a) was at least partly responsible for the age-associated respiration defects found in elderly fibroblasts (Fig. 1a) by inducing a decrease in glycine production and a resultant decrease in mitochondrial translation. This possibility was supported by our observations that adding glycine to the medium for 10 days restored the reduced respiratory function of TIG102 (Supplementary Fig. 6), suggesting that glycine treatment can effectively prevent elderly fibroblasts from expressing age-associated respiration defects.

## Discussion

We reprogrammed human fibroblast lines by generating iPSCs, and showed that the reprogramming of fibroblasts derived from elderly subjects restored age-associated respiration defects. Therefore, these age-associated phenotypes found in elderly fibroblasts are regulated reversibly and are similar to differentiation phenotypes in that both are controlled by epigenetic regulation, not by mutations in either nuclear or mtDNA. Given that human aging can be seen as a consequence of a programmed phenomenon, it is possible that epigenetic regulation also controls human aging. However, further studies are required to generalize the concept that human aging and age-associated disorders—in the same way as the respiration defects found in elderly fibroblasts—are expressed under the control of epigenetic regulation.

We also showed that age-associated mitochondrial respiration defects (Fig. 1a) were expressed in the absence of either ROS overproduction in the mitochondria (Fig. 1b) or the accumulation of somatic mutations in mtDNA (Fig. 1c). One explanation for the absence of an age-associated increase in somatic mutations in mtDNA (Fig. 1c) is the presence of a dynamic balance between the creation and segregation of somatic mutations in mtDNA during repeated cell division. This absence could also be a consequence of the preferential growth of cells possessing mtDNA without somatic mutations during repeated division of the primary fibroblasts obtained by biopsy. Here, however, our focus was on the causes of respiration defects expressed in elderly human fibroblast lines (Fig. 1a), and respiration defects were still expressed even after repeated divisions of cells from the primary biopsy samples. It is therefore likely that these age-associated respiration defects are caused neither by ROS overproduction nor by the accumulation of somatic mutations in mtDNA. Furthermore, even when somatic mutations accumulate in the mtDNA, consequent mitochondrial respiration defects would still be prevented by the exchange of genetic products throughout the mitochondria within a cell^19^,^20^,^21^,^22^. The question that then arises is: What causes age-associated mitochondrial respiration defects by epigenetic regulation?

Our findings revealed that epigenetic downregulation of nuclear-coded genes, including *GCAT* and *SHMT2*, which regulate glycine production in mitochondria^17^,^18^, results in respiration defects. Our previous studies showed that the age-associated respiration defects in elderly fibroblasts^10^ are likely due in part to reduced translation activity in the mitochondria, but not in the cytoplasm^11^. Therefore, defects in glycine metabolism in the mitochondria as a result of a reduction in *SHMT2* and *GCAT* expression would be partly responsible for the reduction in mitochondrial translation, resulting in the expression of age-associated respiration defects. Because continuous glycine treatment restored respiration defects in elderly human fibroblasts (Supplementary Fig. 6), glycine supplementation may be effective in preventing age-associated respiration defects and thus benefiting the health of elderly human subjects. To confirm this hypothesis model mice deficient in *GCAT* or *SHMT2*, or both, would need to be generated to examine whether they expressed respiration defects and premature aging phenotypes and, if so, whether these disorders could be prevented by continuous glycine administration.

Recently, abnormalities of autophagy were proposed to be involved in the dysfunction of organelles, including mitochondria^23^. We showed that no genes were selected from our microarray analysis by using the GO terms, aging and autophagy (Supplementary Fig. 4a), and that no morphological abnormalities developed in the mitochondria of elderly fibroblasts (Supplementary Fig 1b). However, further work is required to examine whether age-associated abnormalities of autophagy in the mitochondria are in fact involved in the expression of age-associated respiration defects and are under the control of epigenetic regulation.

## Methods

### Cells and cell culture

TIG3S, TIG101, TIG102, TIG106, TIG107, TIG118, TIG120, and TIG121 are human diploid fibroblast lines. TIG3S, TIG101, TIG102, TIG107, TIG120, and TIG121 were purchased from the Japanese Collection of Research Bioresources (Tokyo, Japan), and TIG106 and TIG118 were obtained from the Tokyo Metropolitan Institute of Gerontology (Tokyo, Japan). Fibroblasts were grown in Minimum Essential Medium (MEM; Gibco, USA) containing 10% fetal bovine serum (SIGMA, USA) and 1% penicillin/streptomycin (Gibco).

### Measurement of O_2_ consumption rates

Oxygen consumption rates of fibroblasts were estimated in 2 ml PBS by using a YSI model 5331 Clark-type oxygen probe (YSI Incorporated, USA) and Monitor (YSI Model 5300). The reaction chambers were placed in a YSI Model 5301 standard bath assembly and were maintained at 37 °C by water circulation in the bath. PBS was placed in the reaction chambers and the probe was calibrated with PBS before the experiment. The O_2_ consumption rate was calculated as the rate of decrease in O_2_ concentration following the addition of 10^6^ fibroblast cells; it was expressed as % O_2_ consumed per second and then finally normalized by cell volumes. Cell volumes V (mm^3^) were calculated by using the formula for the volume of a sphere,


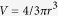


and cell radius r were measured with Image J (NIH Image, USA).

### Measurement of mitochondrial ROS

Generation of mitochondrial ROS (superoxide) was quantitatively estimated by using the mitochondrial superoxide indicator MitoSOX-Red (Invitrogen, USA). Cells were mildly trypsinized and then suspended at a density of 1 × 10^5^ cells/ml in PBS. Next, they were incubated with 5 μM MitoSOX-Red for 15 min at 37 °C in PBS. They were then washed twice with PBS and immediately analyzed with a FACScan flow cytometer (Becton Dickinson, USA). Data were analyzed with FlowJo software (Tree Star, USA).

### Deep sequence analysis of mtDNA

Total cellular DNA (500 ng) extracted from fibroblasts by using a Puregene Core Kit A (Qiagen, USA) was used as template for each PCR reaction. Amplicons generated with eight sets of primers were produced with Takara PrimeSTAR HS DNA polymerase (Takara Bio, Japan) in a reaction volume of 50 μl containing 2.5 mM dNTP mixture, 0.3 μM forward primer, 0.3 μM reverse primer, and PrimeSTAR HS DNA polymerase. The sequences of the primers are shown in Supplementary Table 2. The cycling conditions used were as follows: 1 cycle of 98 °C for 1 min; then 16 cycles of 98 °C for 10 s, 55 °C for 15 s, and 72 °C for 3 min. PCR amplicons were purified with a QIAquick PCR purification kit (Qiagen). Purified DNA was subjected to deep sequencing with a Genome Analyzer IIx (Illumina, USA). Sequencing reads were assembled and compared with the reference sequences (GenBank accession no. AB055387).

### Imaging of mitochondria

Fibroblasts were treated for 30 min at 37 °C with 100 nM Mitotracker Red (Molecular Probes, USA), in accordance with the manufacturer’s instructions, for specific staining of mitochondria in the culture medium. Cells were then fixed with paraformaldehyde for 10 min and stained with 4’,6-diamidino-2-phenylindole, dihydrochloride for nuclear counterstaining. Mitochondrial morphology was analyzed by confocal microscopy (LSM700; Carl Zeiss Microscopy, Germany).

### Measurement of mtDNA copy number

Total mtDNA content was estimated by a real-time PCR technique using a Quantitect Cyber Green Kit (Qiagen) and an ABI PRISM 7900HT sequence detection system (Applied Biosystems, USA). To compare the total mtDNA content, nuclear gene glyceraldehyde-3-phosphate dehydrogenase (GAPDH) was measured as an internal control. The primer set specific for mtDNA was 5′-TACATTACTGCCAGCCACCA-3′ and 5′-GTGGCTTTGGAGTTGCAGTT-3′. The primer set specific for GAPDH was 5′-TACAGGGGTGATGTGGGGAG-3′ and 5′-AGTGATGGCATGGACTGTGG-3′.

### hiPSC generation and redifferentiation

hiPSCs were generated by using episomal vectors, as described previously^14^. Briefly, human diploid fibroblasts cultivated in DMEM supplemented with 10% FBS were transfected with episomal plasmids including the reprogramming gene set (*OCT3/4*, *SOX2*, *KLF4*, *L-MYC*, *LIN28*, and p53shRNA) by using a Neon electroporation system (Life Technologies, USA). After 7 days, the transfected cells were harvested, plated onto 10-cm dishes covered with mitomycin-C-treated mouse embryonic fibroblasts or SNL feeder cells, and then cultured in primate ES cell medium (Gibco) supplemented with 4 ng/ml basic fibroblast growth factor (PEPRO TECH, USA) as described previously^13^. About 4 weeks after the transfection, ES-like cells were collected and expanded on SNL feeder cells for further analysis. For redifferentiation of the hiPSCs into fibroblasts, hiPSCs were induced to form embryoid bodies by culture on gelatin-coated plates. After five passages, most of the cells had a fibroblast-like morphology and were designated as redifferentiated fibroblasts.

### Immunocytochemistry

Immunocytochemistry was performed as described previously^13^. The primary antibodies used were P4HB (Acris Antibodies, USA), SSEA-4 (Millipore, USA), TRA-1-60 (Millipore, USA), and Nanog (Reprocell, Japan). Secondary antibodies were GFP anti-rabbit IgG (Molecular Probes, USA) to detect P4HB, Alexa Fluor 594 anti-mouse IgG (Molecular Probes) to detect SSEA-4 and TRA-1-60, and Alexa Fluor 488 anti-rabbit IgG (Molecular Probes) to detect Nanog.

### Microarray and data analysis

Microarray experiments were performed with an Agilent Expression Array Whole Human Genome Oligo DNA microarray (Agilent Technologies, USA) at the Takara-Bio Corporation (Shiga, Japan), with the microarray service certificated by Agilent Technologies. Total RNA was extracted from each fibroblast line in accordance with the manufacturer’s protocol. Treatment with DNase I was conducted to eliminate genomic DNA contamination. The quality of the extracted RNA was assessed with a BioAnalyzer 2100 system (Agilent Technologies). The RNA was then Cy3-labeled by using a Low Input Quick Amp Labeling Kit (Agilent Technologies). Labeled cRNA samples were hybridized to a SurePrint G3 Human GE 8 × 60 K v2 Microarray (Agilent Technologies) at 65 °C for 17 h by using a Gene Expression Hybridization Kit (Agilent Technologies). After hybridization, the microarrays were washed and scanned with an Agilent microarray Scanner. Signal intensities were evaluated with Agilent Feature Extraction software. Microarray data analysis was performed with the R software and Bioconductor packages.

### Real-time quantitative PCR

Total RNA was extracted from human fibroblast cells by using ISOGEN reagent (Nippon Gene, Japan). RNA samples were subjected to DNase I treatment (Invitrogen) to eliminate DNA contaminants and reverse transcribed by using Oligo (dT)_12-18_ primer, 10 mM dNTP Mix, 0.1 M DTT, RNase Out Recombinant Ribonuclease Inhibitor, and SuperScript II-Reverse Transcriptase (Invitrogen). cDNA samples were subjected to RNase H treatment (Invitrogen), and applied to the real-time PCR reaction. Real-time monitoring PCR was performed with SYBR Green PCR Master Mix (Qiagen) and an ABI PRISM 7900HT sequence detection system (Applied Biosystems). The relative mRNA expression was quantified by using the comparative ΔΔCT method. The relative expression level of each gene was represented as a cycle threshold (CT). The normalized expression level was then calculated as ΔCT = CT (target gene) – CT (control gene). Differential expression was calculated as ΔΔCT = ΔCT (target sample) – ΔCT (control sample). Fold change was calculated as 2^–ΔΔCT^. *UBC* was used as an internal control. The sequences of the primers are shown in Supplementary Table 3.

### Construction of lentiviral vectors

Human GCAT cDNA was provided by the RIKEN Bio-Resource Center (BRC) through the National Bio-Resource Project of the MEXT, Japan^24^,^25^,^26^,^27^. The GCAT cDNA was amplified by PCR using the full-length cDNA clone IRAL03F16 (RIKEN BRC) with the following primers: 5′-CACCATGTGGCCTGGGAACGCC-3′ (forward) and 5′-TCAGGGCAGTGCCCCGTG-3′ (reverse). The PCR-amplified cDNA was inserted into the pENTR/D-TOPO entry vector plasmid (Invitrogen) and verified by DNA sequencing. The GCAT cDNA in pENTR/D-TOPO was then transferred to the CSII-EF-RfA-IRES-Puro lentiviral vector plasmid by using the Gateway LR clonase (Invitrogen), resulting in CSII-EF-GCAT-IRES-Puro. The shRNA sequence targeting human GCAT mRNA, CCTTAACTTCTGTGCCAACAA, and the scrambled sequence were inserted into pENTR4-H1tetOx1 entry vector plasmid (RIKEN BRC) and then transferred to the CS-RfA-CG lentiviral vector plasmid by using Gateway LR clonase, resulting in CS-GCAT-shRNA-CG and CS-Scramble-shRNA-CG. Recombinant lentiviral vector expressing GCAT cDNA or shRNA was produced by transient transfection of three plasmids, pCAG-HIVgp, pCMV-VSV-G-RSV-Rev, and the lentiviral vector plasmid (CSII-EF-GCAT-IRES-Puro or CS-GCAT-shRNA-CG or CS-Scramble-shRNA-CG), into 293 T cells. Culture supernatant containing lentiviral vector was concentrated by ultracentrifugation (50,000 × g for 2 hours at 20 °C), and the viral pellet was resuspended in Hank’s balanced salt solution. The titer of vectors was determined by infection of HeLa cells with serial dilutions of the vector stocks followed by measurement of puromycin-resistant cells for cDNA vector and FACS analysis for GFP + cells for shRNA vector.

### siRNAs and transfection

Stealth RNAi oligonucleotides were used for siRNA experiments (Invitrogen). The SHMT2 Stealth Select RNAi oligonucleotide (Target Accession No. NM_001166356) was used for knockdown of *SHMT2*. The Stealth RNAi negative control Duplex (Invitrogen) was used as a scramble oligonucleotide. The Stealth RNAi oligonucleotides were transfected into the fibroblasts by using Lipofectamine RNAiMAX transfection reagent (Invitrogen) in accordance with the manufacturer’s protocols (15 μl Stealth RNAi oligonucleotide and 30 μl Lipofectamine RNAiMAX in 10 ml MEM).

### Statistical analysis

Results are expressed as means ± SD; probability values <0.05 were considered statistically significant. Statistical analysis of the data was done with the two-tailed unpaired Student’s *t*-test.

## Additional Information

**How to cite this article**: Hashizume, O. *et al.* Epigenetic regulation of the nuclear-coded GCAT and SHMT2 genes confers human age-associated mitochondrial respiration defects. *Sci. Rep.* **5**, 10434; doi: 10.1038/srep10434 (2015).

## Supplementary Material

Supplementary Information
