# Corrigendum: Epigenetic regulation of the nuclear-coded GCAT and SHMT2 genes confers human age-associated mitochondrial respiration defects

**DOI:** 10.1038/srep14591

**Published:** 2015-10-05

**Authors:** Osamu Hashizume, Sakiko Ohnishi, Takayuki Mito, Akinori Shimizu, Kaori Ishikawa, Kazuto Nakada, Manabu Soda, Hiroyuki Mano, Sumie Togayachi, Hiroyuki Miyoshi, Keisuke Okita, Jun-Ichi Hayashi

Scientific Reports 5: Article number: 1043410.1038/srep10434; published online: 05222015; updated: 10052015

The original version of this Article contained a typographical error in the spelling of the author Kaori Ishikawa, which was incorrectly given as Kaori Iashikawa. This has now been corrected in both the PDF and HTML versions of the Article.

