# Supplementary material for: Epigenetic regulation of the nuclear-coded GCAT and SHMT2 genes confers human age-associated mitochondrial respiration defects
Source: Sci Rep. 2015 May 22;5:10434. doi: 10.1038/srep10434 (PMC5377050; doi:10.1038/srep10434)
Supplement: Supplementary Information [file srep10434-s1.pdf]

Supplementary Information for

Epigenetic regulation of the nuclear-coded *GCAT* and *SHMT2* genes confers human age-associated mitochondrial respiration defects

Osamu Hashizume, Sakiko Ohnishi, Takayuki Mito, Akinori Shimizu, Kaori Ishikawa, Kazuto Nakada, Manabu Soda, Hiroyuki Mano, Sumie Togayachi, Hiroyuki Miyoshi, Keisuke Okita & Jun-Ichi Hayashi

Supplementary figure legends

Supplementary Figure 1. Effects of aging on mtDNA copy number and morphology of mitochondria. (a) Comparison of mtDNA copy number between young and elderly groups. The young group (black bars) consisted of the fibroblast lines TIG3S (fetus), TIG121 (age 8 months), TIG120 (6 years), and TIG118 (12 years). The elderly group (open bars) consisted of TIG106 (80 years), TIG107 (81 years), TIG101 (86 years), and TIG102 (97 years). 'Average' indicates the average mtDNA copy number of four fibroblast lines. (b) Comparison of mitochondrial morphologies. Fibroblasts were treated with Mitotracker Red to stain mitochondria and 4',6-diamidino-2-phenylindole (DAPI) to stain nuclei. The higher intensity of mitochondrial staining with Mitotracker Red reflects the higher membrane potential and higher respiratory function in TIG3S (fetus) than in TIG107 (81 years). Bars, 20 μm.

Supplementary Figure 2. Quantitative estimation of mutation frequency at each nucleotide position of mtDNA from eight fibroblast lines by deep sequence analysis. Red and blue dots represent the frequencies of mutations at each nucleotide position of mtDNA from the young group and the elderly group, respectively. Graphs show mutation frequency versus mutation frequency rank. A higher rank represents a higher frequency of the mutation at each nucleotide position. The young group consists of TIG3S (fetus), TIG121 (8 months), TIG120 (6 years), and TIG118 (12 years). The elderly group consists of TIG106 (80 years), TIG107 (81 years), TIG101 (86 years), and TIG102 (97 years).

Supplementary Figure 3. Characterization of human induced pluripotent stem cells isolated from the fibroblast lines TIG3S (fetus), TIG121 (age 8 months), TIG107 (81 years), and TIG102 (97 years). (a) Small colonies with flat embryonic-stem-cell-like morphology were obtained about 4 weeks after transfection with the gene set *OCT3/4*, *SOX2*, *KFL4*, *L-MYC*, *LIN28*, and *p53shRNA*. (b) Examination of pluripotency marker genes by using antibodies to Nanog, TRA-1-60, and SSEA4. Expression of Nanog is shown in red (Alexa Fluor 488), and that of TRA-1-60 or SSEA4 is shown in green (Alexa Fluor 594). Bars, 100 μm.

Supplementary Figure 4. Selection of six nuclear-coded genes associated with age-associated mitochondrial respiration defects in human fibroblasts. (a) Schematic representation of the process of selection of nuclear-coded genes. From 27,958 genes on the microarray, 1446 genes were selected by using the gene ontology (GO) term ‘mitochondria’. From those 1446 genes, 144, 28, 128, and 71 genes were selected by using the GO terms ‘respiration’, ‘aging’, ‘translation’, and ‘amino acids’, respectively. Finally, six genes that were commonly more than 1.5-fold upregulated or downregulated in fibroblast lines from elderly subjects were selected by microarray analysis (solid lines). To examine aging phenotypes other than mitochondrial respiration defects, we used the GO terms ‘aging’ and ‘autophagy’. We then searched for genes that were commonly more than 1.5-fold upregulated or downregulated in fibroblast lines from elderly subjects (broken lines). No genes were selected in this part of the

microarray analysis. (b) Heatmaps depicting transcripts of genes associated with respiration, aging, translation, and amino acids in mitochondria. Values represent log_2 ratios of the average array signals of TIG107 and TIG102 compared with those of TIG3S and TIG121 (TIG107/TIG3S, TIG102/TIG3S, TIG107/TIG121 and TIG102/TIG121). Genes down- and upregulated in the fibroblast lines from elderly subjects (TIG107 and TIG102) are shown in red and green, respectively.

Supplementary Figure 5. Comparison of mRNA levels of the candidate six genes in the young and elderly groups. The six gene candidates for regulation of age-associated respiration defects were selected by using gene ontology terms and a microarray heatmap (Supplementary Fig. 4b). The young group consisted of fibroblast lines TIG3S (fetus), TIG121 (age 8 months), TIG120 (6 years), and TIG118 (12 years). The elderly group consisted of lines TIG106 (80 years), TIG107 (81 years), TIG101 (86 years), and TIG102 (97 years). Levels of transcripts were normalized against UBC expression. Of the six genes examined, age-associate regulation was confirmed to be present in MRPL28, EHHADH, and GCAT.

Supplementary Figure 6. Effects of glycine treatment on the mitochondrial respiratory function of TIG102.

Glycine (140 μM) was included in the medium for 10 days before the estimation of O_2 consumption rates. Experiments were performed in triplicate; error bars indicate ± SD. $*P < 0.05$.

Supplementary Figure 1

a

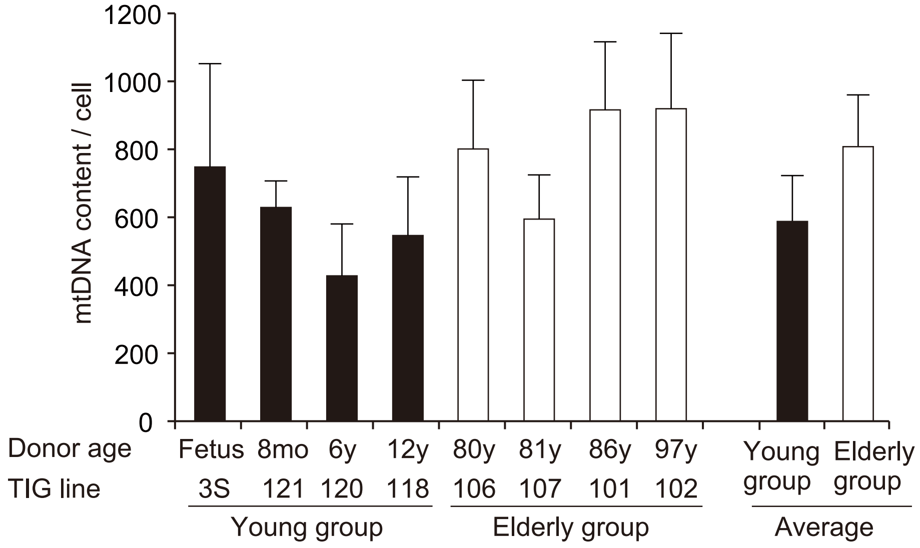

b

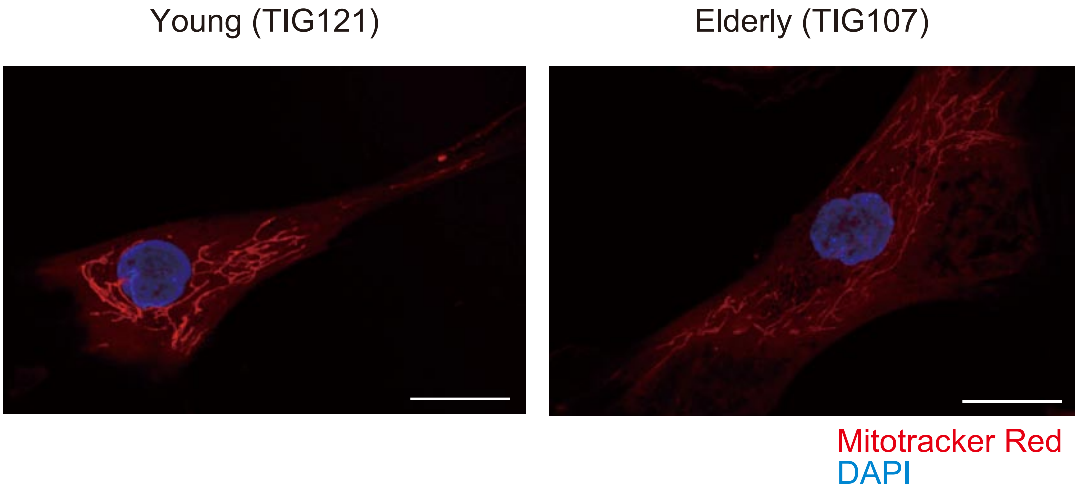

Supplementary Figure 2

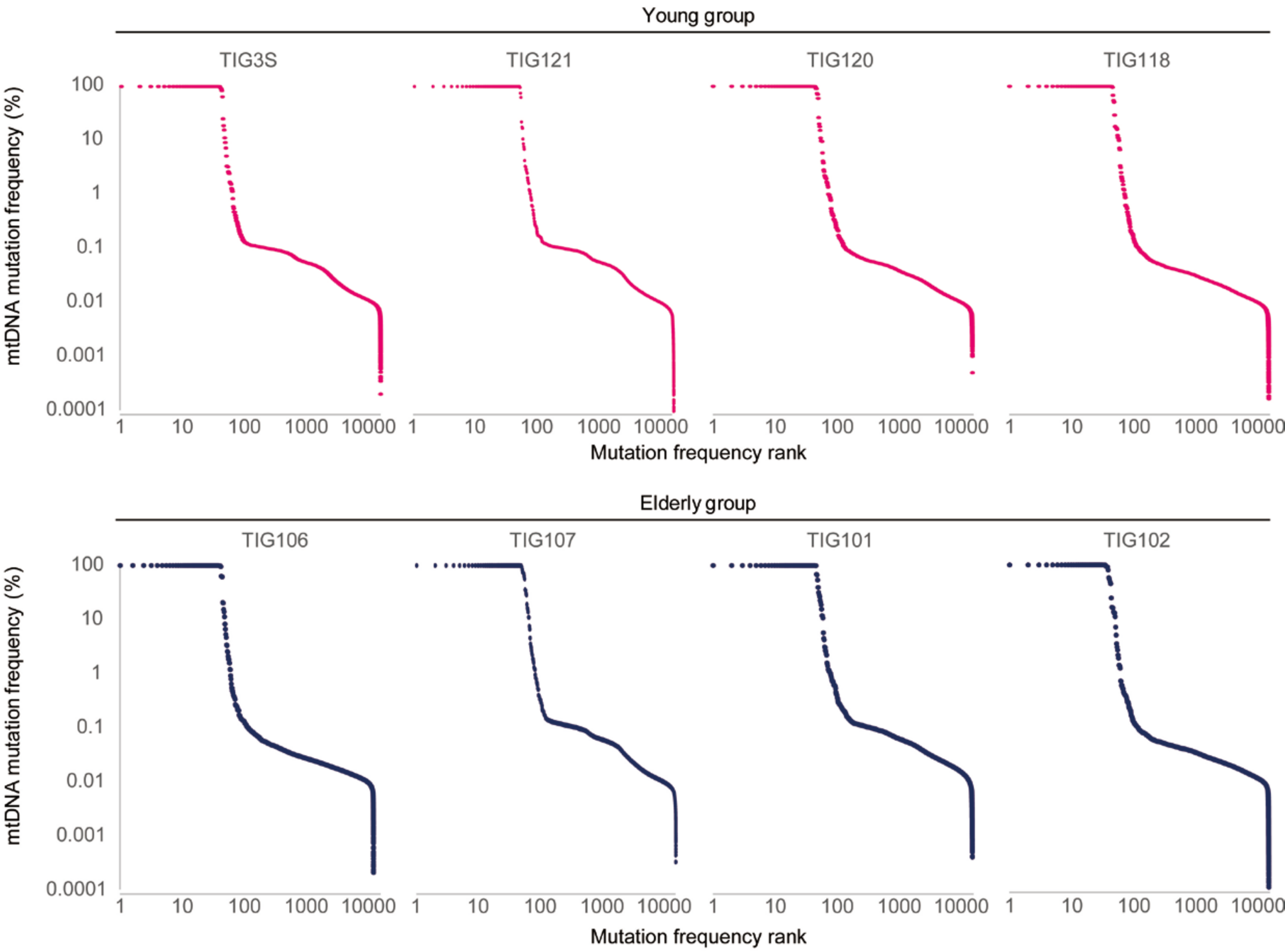

Supplementary Figure 3

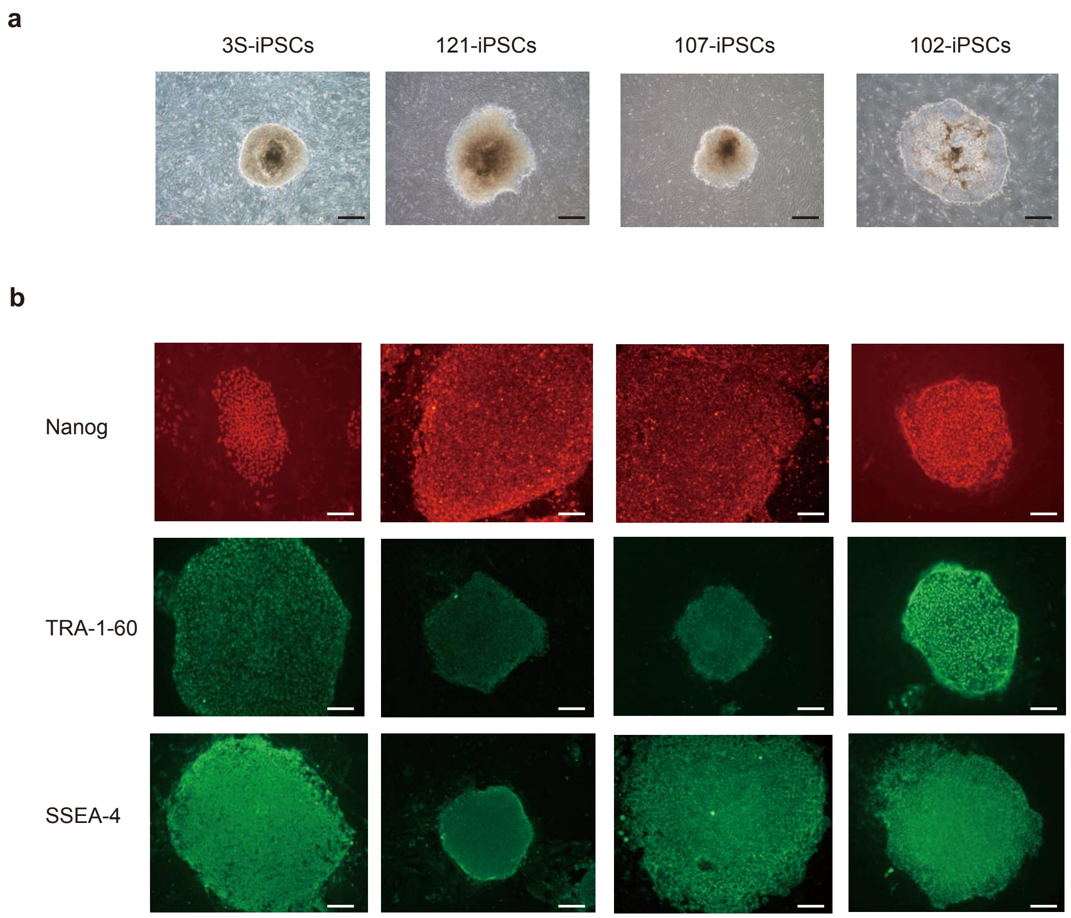

Supplementary Figure 4

a

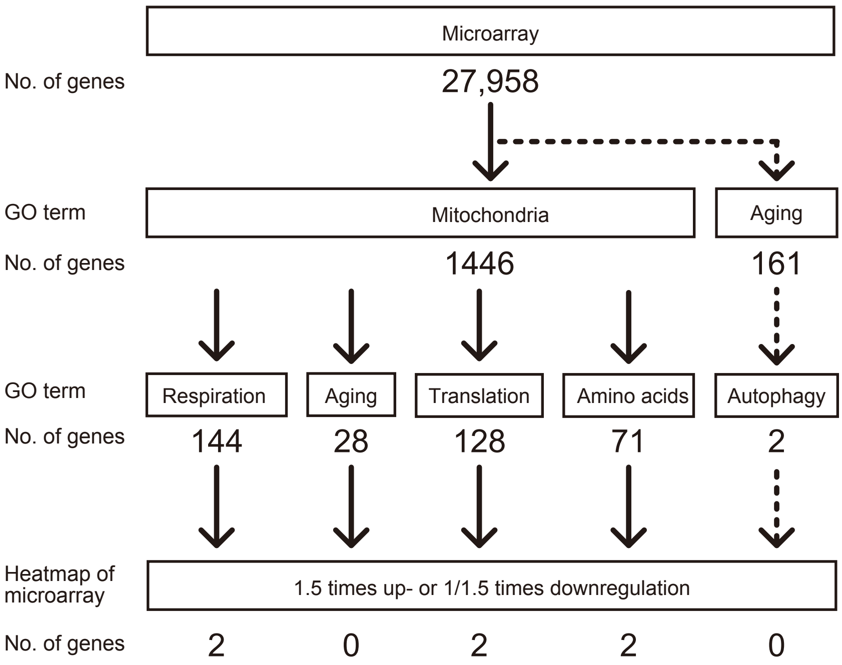

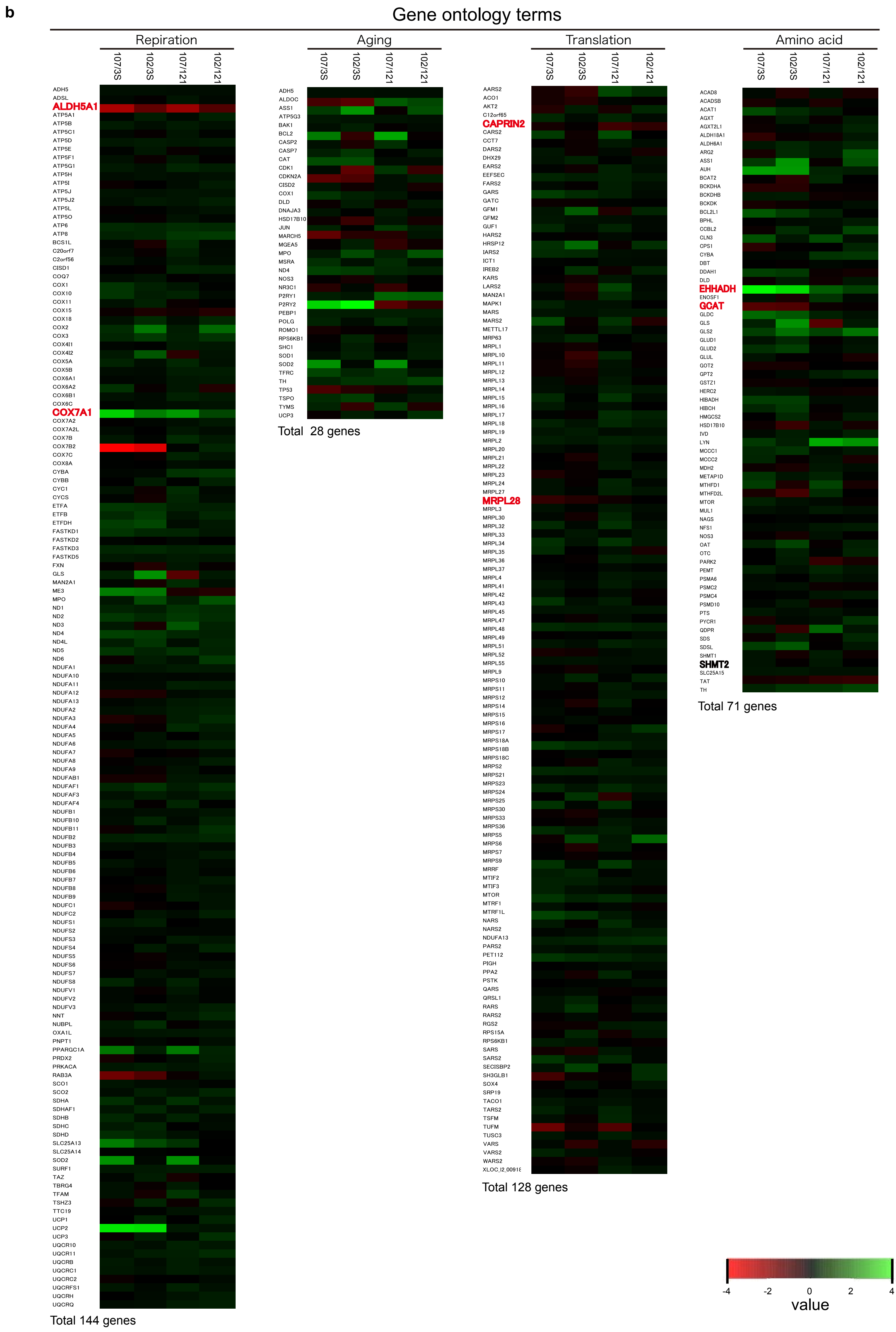
b
Gene ontology terms
Repiration
107/3S
102/3S
107/121
102/121
ADH5
ADSL
ALDH5A1
ATP5A1
ATP5B
ATP5C1
ATP5D
ATP5E
ATP5F1
ATP5G1
ATP5H
ATP5I
ATP5J
ATP5J2
ATP5L
ATP5O
ATP6
ATP8
BCS1L
C20orf7
C2orf56
CISD1
COQ7
COX1
COX10
COX11
COX15
COX18
COX2
COX3
COX4I1
COX4I2
COX5A
COX5B
COX6A1
COX6A2
COX6B1
COX6C
COX7A1
COX7A2
COX7A2L
COX7B
COX7B2
COX7C
COX8A
CYBA
CYBB
CYC1
CYCS
ETFA
ETFB
ETFDH
FASTKD1
FASTKD2
FASTKD3
FASTKD5
FXN
GLS
MAN2A1
ME3
MPO
ND1
ND2
ND3
ND4
ND4L
ND5
ND6
NDUFA1
NDUFA10
NDUFA11
NDUFA12
NDUFA13
NDUFA2
NDUFA3
NDUFA4
NDUFA5
NDUFA6
NDUFA7
NDUFA8
NDUFA9
NDUFAB1
NDUFAF1
NDUFAF3
NDUFAF4
NDUFB1
NDUFB10
NDUFB11
NDUFB2
NDUFB3
NDUFB4
NDUFB5
NDUFB6
NDUFB7
NDUFB8
NDUFB9
NDUFC1
NDUFC2
NDUFS1
NDUFS2
NDUFS3
NDUFS4
NDUFS5
NDUFS6
NDUFS7
NDUFS8
NDUFV1
NDUFV2
NDUFV3
NNT
NUBPL
OXA1L
PNPT1
PPARGC1A
PRDX2
PRKACA
RAB3A
SCO1
SCO2
SDHA
SDHAF1
SDHB
SDHC
SDHD
SLC25A13
SLC25A14
SOD2
SURF1
TAZ
TBRG4
TFAM
TSHZ3
TTC19
UCP1
UCP2
UCP3
UQCR10
UQCR11
UQCRB
UQCRC1
UQCRC2
UQCRFS1
UQCRH
UQCRQ
Total 144 genes
Aging
107/3S
102/3S
107/121
102/121
ADH5
ALDOC
ASS1
ATP5G3
BAK1
BCL2
CASP2
CASP7
CAT
CDK1
CDKN2A
CISD2
COX1
DLD
DNAJA3
HSD17B10
JUN
MARCH5
MGEA5
MPO
MSRA
ND4
NOS3
NR3C1
P2RY1
P2RY2
PEBP1
POLG
ROMO1
RPS6KB1
SHC1
SOD1
SOD2
TFRC
TH
TP53
TSPO
TYMS
UCP3
Total 28 genes
Translation
107/3S
102/3S
107/121
102/121
AARS2
ACO1
AKT2
C12orf65
CAPRIN2
CARS2
CCT7
DARS2
DHX29
EARS2
EEFSEC
FARS2
GARS
GATC
GFM1
GFM2
GUF1
HARS2
HRSP12
IARS2
ICT1
IREB2
KARS
LARS2
MAN2A1
MAPK1
MARS
MARS2
METTL17
MRP63
MRPL1
MRPL10
MRPL11
MRPL12
MRPL13
MRPL14
MRPL15
MRPL16
MRPL17
MRPL18
MRPL19
MRPL2
MRPL20
MRPL21
MRPL22
MRPL23
MRPL24
MRPL27
MRPL28
MRPL3
MRPL30
MRPL32
MRPL33
MRPL34
MRPL35
MRPL36
MRPL37
MRPL4
MRPL41
MRPL42
MRPL43
MRPL45
MRPL47
MRPL48
MRPL49
MRPL51
MRPL52
MRPL55
MRPL9
MRPS10
MRPS11
MRPS12
MRPS14
MRPS15
MRPS16
MRPS17
MRPS18A
MRPS18B
MRPS18C
MRPS2
MRPS21
MRPS23
MRPS24
MRPS25
MRPS30
MRPS33
MRPS36
MRPS5
MRPS6
MRPS7
MRPS9
MRRF
MTIF2
MTIF3
MTOR
MTRF1
MTRF1L
NARS
NARS2
NDUFA13
PARS2
PET112
PIGH
PPA2
PSTK
QARS
QRSL1
RARS
RARS2
RGS2
RPS15A
RPS6KB1
SARS
SARS2
SECISBP2
SH3GLB1
SOX4
SRP19
TACO1
TARS2
TSFM
TUFM
TUSC3
VARS
VARS2
WARS2
Total 128 genes
Amino acid
107/3S
102/3S
107/121
102/121
ACAD8
ACADSB
ACAT1
AGXT
AGXT2L1
ALDH18A1
ALDH6A1
ARG2
ASS1
AUH
BCAT2
BCKDHA
BCKDHB
BCKDK
BCL2L1
BPHL
CCBL2
CLN3
CPS1
CYBA
DBT
DDAH1
DLD
EHHADH
ENOSF1
GCAT
GLDC
GLS
GLS2
GLUD1
GLUD2
GLUL
GOT2
GPT2
GSTZ1
HERC2
HIBADH
HIBCH
HMGCS2
HSD17B10
IVD
LYN
MCCC1
MCCC2
MDH2
METAP1D
MTHFD1
MTHFD2L
MTOR
MUL1
NAGS
NFS1
NOS3
OAT
OTC
PARK2
PEMT
PSMA6
PSMC2
PSMC4
PSMD10
PTS
PYCR1
QDPR
SDS
SDSL
SHMT1
SHMT2
SLC25A15
TAT
TH
Total 71 genes
-4
-2
0
2
4
value

Supplementary Figure 5

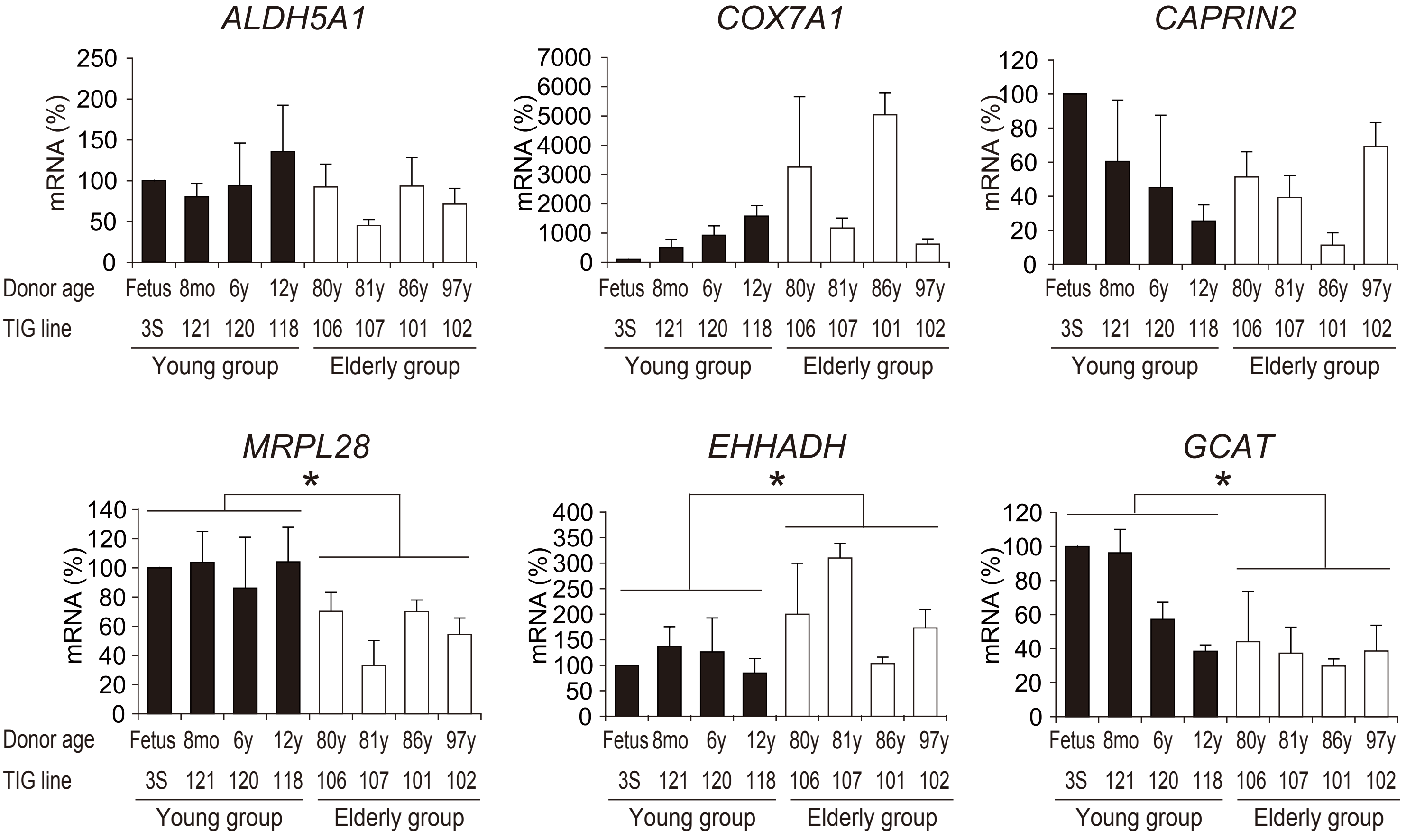

Supplementary Figure 6

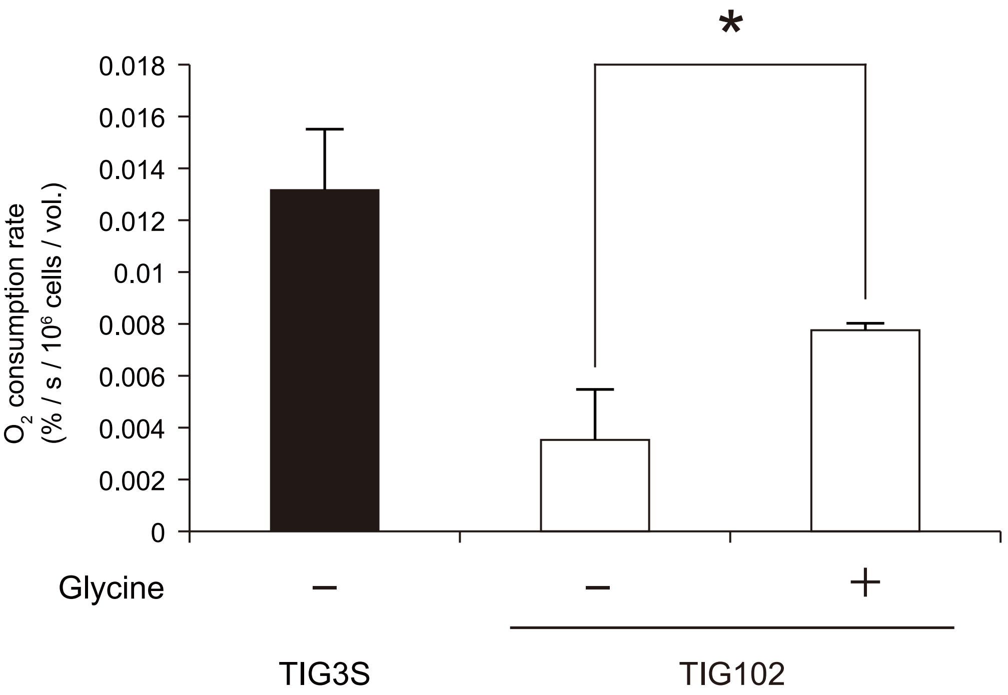

Table S1. Characterization of the frequent mutations existing in more than 90% mtDNA revealed by the deep sequence analysis of eight fibroblast lines.

Human fibroblast lines	Position[a]	Gene	Ref[a]	Alteration	Related disease[b]	Total read number	Alteration read number	Alteration ratio (%)	Indel type	Amino acid change
TIG3S	103	D-loop	A	G	polymorphism	989611	989349	99.9608	-	-
(fetus)	199		C	T	polymorphism	448971	448801	99.9485	-	-
	204		C	T	polymorphism	479987	479793	99.9548	-	-
	235		A	G	polymorphism	193956	193791	99.9056	-	-
	660	RNR1	A	G	polymorphism	987308	987028	99.945	-	-
	706		A	G	polymorphism	993523	993174	99.9582	-	-
	951		C	CA	polymorphism	999511	914384	91.4831	1-ins	-
	1594		A	G	polymorphism	986159	985913	99.9562	-	-
	1732	RNR2	A	G	polymorphism	987965	987835	99.974	-	-
	2146		T	TA	polymorphism	992672	921618	92.8421	1-ins	-
	4243	ND1	T	C	polymorphism	988320	987689	99.9188	-	-
	4650	ND2	G	A	polymorphism	988665	988501	99.9764	-	-
	4819		A	G	polymorphism	982588	982073	99.9334	-	T-A
	6570	COX1	A	AG	polymorphism	907920	838304	92.3324	1-ins	-
	8548	ATP8/ATP6	A	G	polymorphism	779011	778469	99.9173	-	T-A (ATP6)
	8569	ATP6	A	G	polymorphism	779299	778962	99.942	-	-
	8769		G	A	polymorphism	482303	482194	99.9677	-	-
	8779		C	T	polymorphism	481057	480879	99.9555	-	H-Y
	8814		T	C	polymorphism	453667	453547	99.9343	-	-
	9525	COX3	C	T	polymorphism	990849	990737	99.9771	-	-
	9935		C	T	polymorphism	987933	987799	99.9761	-	-
	10383	ND3	G	A	polymorphism	988254	988089	99.9767	-	-
	11131	ND4	T	C	polymorphism	986472	986334	99.9285	-	-
	11521		C	T	polymorphism	991976	991782	99.9626	-	-
	11632		C	T	polymorphism	992308	933903	94.1044	-	-
	11899		A	G	polymorphism	993864	992982	99.9026	-	-
	12177	TRNH	A	G	polymorphism	989387	989033	99.9514	-	-
	12346	ND5	G	A	polymorphism	986936	986789	99.975	-	-
	12690		C	T	polymorphism	989901	989710	99.9654	-	-
	14079		T	C	polymorphism	983826	983702	99.9655	-	-
	14455	ND6	C	T	polymorphism	932595	932454	99.9725	-	-
	14751	CYTB	C	T	polymorphism	889483	889224	99.96	-	T-I
	15208		T	C	polymorphism	985048	984884	99.9677	-	-
	15493		T	C	polymorphism	936525	936387	99.9706	-	-
	15647		G	A	polymorphism	986318	986212	99.9805	-	I-V
	15836		G	A	polymorphism	991624	991515	99.983	-	I-V

	15912	TRNT	A	G	polymorphism	992369	992078	99.9128	-	-
	16125	D-loop	C	T	polymorphism	394184	394144	99.9827	-	-
	16172		C	T	polymorphism	273	272	99.6337	-	-
	16174		C	T	polymorphism	1191	1085	91.0999	-	-
	16228		C	T	polymorphism	26986	26786	99.2552	-	-
	16250		G	A	polymorphism	26421	26270	99.4247	-	-
	16275		C	T	polymorphism	513353	513218	99.9601	-	-
	16303		T	A	polymorphism	999765	999669	99.9802	-	-
	16504		C	T	polymorphism	356511	356394	99.9512	-	-
total numbers of alterations										
45										
TIG121	103	D-loop	A	G	polymorphism	940020	939710	99.9522	-	-
(8m)	152		T	C	polymorphism	247388	247174	99.7797	-	-
	199		C	T	polymorphism	732147	731982	99.9638	-	-
	204		C	T	polymorphism	819782	819624	99.9756	-	-
	488		T	C	polymorphism	629931	629833	99.977	-	-
	564		A	AC	polymorphism	724031	652264	90.0879	1-ins	-
	706	RNR1	A	G	polymorphism	993785	993336	99.9467	-	-
	951		C	CA	polymorphism	999434	920561	92.1082	1-ins	-
	1594		A	G	polymorphism	986920	986643	99.9547	-	-
	2242	RNR2	A	G	polymorphism	981446	981305	99.9715	-	-
	3006		G	A	polymorphism	989735	989603	99.9784	-	-
	4878	ND2	C	T	polymorphism	993953	955904	96.1541	-	-
	5173		C	A	polymorphism	977007	976586	99.9444	-	L-M
	6569	COX1	A	AG	polymorphism	625743	577879	92.3359	1-ins	-
	6570		A	AG	polymorphism	625844	577879	92.3359	1-ins	-
	8014	COX2	G	A	polymorphism	856617	856501	99.9756	-	-
	8399	ATP8	C	T	polymorphism	908491	908348	99.9711	-	L-F
	8435		T	C	polymorphism	863569	863464	99.9763	-	-
	8569	ATP6	A	G	polymorphism	653775	653506	99.944	-	-
	8686		A	G	polymorphism	910866	910715	99.9648	-	T-A
	8769		G	A	polymorphism	872371	872274	99.9846	-	-
	8814		T	C	polymorphism	831673	831601	99.9752	-	-
	9935	COX3	C	T	polymorphism	988570	988431	99.9763	-	-
	10166	ND3	C	T	polymorphism	964873	964676	99.9645	-	-
	10385		C	T	polymorphism	989721	989562	99.9765	-	-
	10858	ND4	T	C	polymorphism	882227	882032	99.9095	-	-
	11131		T	C	polymorphism	988894	988761	99.9278	-	-
	11899		A	G	polymorphism	992936	991561	99.8542	-	-
	12177	TRNH	A	G	polymorphism	990454	990172	99.9582	-	-
	12346	ND5	G	A	polymorphism	985959	985817	99.9788	-	-
	12690		C	T	polymorphism	988911	988692	99.9633	-	-

	13812		A	G	polymorphism	996116	995899	99.965	-	-
	14076		A	G	polymorphism	993980	993813	99.969	-	-
	14165	ND6	T	C	polymorphism	995551	995383	99.969	-	Y-C
	14455		C	T	polymorphism	993200	993010	99.9669	-	-
	14653		C	T	polymorphism	990390	990282	99.9751	-	-
	14751	CYTB	C	T	polymorphism	989931	989806	99.9799	-	T-I
	14768		T	C	polymorphism	992970	992893	99.9788	-	-
	14912		A	G	polymorphism	990606	990300	99.9538	-	T-A
	15028		G	A	polymorphism	985795	985681	99.9844	-	-
	15202		G	A	polymorphism	823110	822980	99.9803	-	-
	15208		T	C	polymorphism	849230	849109	99.97	-	-
	15286		G	A	polymorphism	989654	989514	99.9807	-	-
	15425		T	C	polymorphism	988911	988778	99.9433	-	-
	15493		T	C	polymorphism	978494	978373	99.9761	-	-
	15647		G	A	polymorphism	981377	981274	99.9798	-	I-V
	15790		A	G	polymorphism	985755	985560	99.9431	-	I-V
	15836		G	A	polymorphism	966041	965932	99.9854	-	-
	15912	TRNT	A	G	polymorphism	991096	990847	99.9208	-	-
	15936		A	G	polymorphism	990670	990286	99.9497	-	-
	16125	D-loop	C	T	polymorphism	436189	436141	99.9819	-	-
	16228		C	T	polymorphism	856600	856249	99.9505	-	-
	16250		G	A	polymorphism	998999	998821	99.9791	-	-
	16303		T	A	polymorphism	818137	818035	99.9808	-	-
	16347		T	C	polymorphism	999105	998946	99.9705	-	-
	16504		C	T	polymorphism	621330	621163	99.9625	-	-
total numbers of alterations 56										
TIG120 (6y)	103	D-loop	A	G	polymorphism	929806	929498	99.9568	-	-
	194		C	T	polymorphism	10159	10066	99.0255	-	-
	204		C	T	polymorphism	165091	164897	99.8728	-	-
	207		G	A	polymorphism	205408	205156	99.8569	-	-
	488		T	C	polymorphism	648509	648416	99.9685	-	-
	706	RNR1	A	G	polymorphism	978925	978195	99.9152	-	-
	951		C	CA	polymorphism	999326	948397	94.9037	1-ins	-
	1594		A	G	polymorphism	987571	987333	99.9611	-	-
	2762	RNR2	C	T	polymorphism	995461	898728	90.2742	-	-
	3006		G	A	polymorphism	989846	989678	99.9715	-	-
	3386	ND1	G	A	polymorphism	705795	705683	99.9751	-	-
	5173	ND2	C	A	polymorphism	949650	949386	99.9615	-	L-M
	5257		G	A	polymorphism	788823	788526	99.9539	-	A-T
	6570	COX1	A	AG	polymorphism	233902	214724	91.8008	1-ins	-
	6713		T	C	polymorphism	316024	315686	99.8744	-	-

	8399	ATP8	C	T	polymorphism	872684	872515	99.9695	-	-
	8569	ATP6	A	G	polymorphism	550502	550227	99.9321	-	-
	8686		A	G	polymorphism	936514	936378	99.9676	-	T-A
	8769		G	A	polymorphism	818207	818114	99.9852	-	-
	8814		T	C	polymorphism	736098	736030	99.9666	-	-
	9740	COX3	G	A	polymorphism	989712	989609	99.9857	-	-
	9935		C	T	polymorphism	992238	992061	99.9713	-	-
	10385	ND3	C	T	polymorphism	990176	990024	99.9766	-	-
	10858	ND4	T	C	polymorphism	875106	874895	99.936	-	-
	11131		T	C	polymorphism	984715	984595	99.9563	-	-
	11899		A	G	polymorphism	992580	989768	99.7104	-	-
	12177	TRNH	A	G	polymorphism	990805	990554	99.9637	-	-
	12346	ND5	G	A	polymorphism	825073	824959	99.9778	-	-
	12690		C	T	polymorphism	976980	976679	99.9538	-	-
	14113		A	G	polymorphism	983559	891780	90.6504	-	T-A
	14455	ND6	C	T	polymorphism	868399	868243	99.9703	-	-
	14653		C	T	polymorphism	988564	988448	99.9769	-	-
	14751	CYTB	C	T	polymorphism	908569	908449	99.9799	-	T-I
	14768		T	C	polymorphism	894998	894908	99.9773	-	-
	15028		G	A	polymorphism	986890	986767	99.9795	-	-
	15208		T	C	polymorphism	995346	995221	99.9729	-	-
	15286		G	A	polymorphism	970827	970693	99.9795	-	-
	15493		T	C	polymorphism	922508	922399	99.9736	-	-
	15647		G	A	polymorphism	986148	986048	99.9843	-	I-V
	15836		G	A	polymorphism	990550	990428	99.9833	-	I-V
	15912	TRNT	A	G	polymorphism	993139	992854	99.9397	-	-
	16125	D-loop	C	T	polymorphism	432780	432739	99.9834	-	-
	16208		T	C	polymorphism	1311	1309	99.6949	-	-
	16228		C	T	polymorphism	246	240	97.561	-	-
	16230		C	T	polymorphism	806	802	99.3797	-	-
	16250		G	A	polymorphism	6104	6061	99.2628	-	-
	16303		T	A	polymorphism	26023	25964	99.7617	-	-
	16304		A	G	polymorphism	39800	39579	99.407	-	-
	16347		T	C	polymorphism	999073	998826	99.9644	-	-
	16504		C	T	polymorphism	599188	599037	99.97	-	-
total numbers of alterations										
50										
TIG118	103	D-loop	A	G	polymorphism	952137	951846	99.9615	-	-
(12y)	152		T	C	polymorphism	385500	385296	99.8895	-	-
	199		C	T	polymorphism	592022	591878	99.9644	-	-
	204		C	T	polymorphism	658146	658021	99.9745	-	-
	488		T	C	polymorphism	766339	766242	99.9687	-	-

706	RNR1	A	G	polymorphism	993751	993379	99.9544	-	-
951		C	CA	polymorphism	999397	920827	92.1383	1-ins	-
1594		A	G	polymorphism	986481	986233	99.9591	-	-
3006	RNR2	G	A	polymorphism	990826	990673	99.9785	-	-
3201		C	T	polymorphism	995149	994624	99.9306	-	-
4878	ND2	C	T	polymorphism	989346	946222	95.6237	-	-
5173		C	A	polymorphism	972540	972054	99.936	-	L-M
6493	COX1	C	A	polymorphism	536062	536007	99.9744	-	L-I
6570		A	AG	polymorphism	544580	511337	93.8957	1-ins	-
8399	ATP8	C	T	polymorphism	847721	847609	99.9712	-	L-F
8458		T	C	polymorphism	746134	745328	99.8506	-	-
8569	ATP6	A	G	polymorphism	716878	716588	99.9382	-	-
8686		A	G	polymorphism	971753	971595	99.9692	-	T-A
8769		G	A	polymorphism	930365	930249	99.9815	-	-
8814		T	C	polymorphism	889432	889360	99.9728	-	-
9935	COX3	C	T	polymorphism	988228	988067	99.9713	-	-
10385	ND3	C	T	polymorphism	989552	989389	99.9759	-	-
10395	TRNR	T	C	polymorphism	991178	991001	99.9672	-	-
10858	ND4	T	C	polymorphism	892005	891816	99.9434	-	-
11131		T	C	polymorphism	987213	987083	99.9583	-	-
11899		A	G	polymorphism	993989	992580	99.8507	-	-
12177	TRNH	A	G	polymorphism	990032	989773	99.965	-	-
12346	ND5	G	A	polymorphism	978662	978536	99.9788	-	-
12690		C	T	polymorphism	990883	990661	99.9636	-	-
12960		A	G	polymorphism	966667	966397	99.9605	-	-
14455	ND6	C	T	polymorphism	967419	967243	99.9674	-	-
14653		C	T	polymorphism	992032	991933	99.9771	-	-
14751	CYTB	C	T	polymorphism	946269	946131	99.9758	-	T-I
14768		T	C	polymorphism	969862	969758	99.9748	-	-
14964		T	C	polymorphism	990686	990530	99.9734	-	-
15028		G	A	polymorphism	987942	987829	99.9818	-	-
15208		T	C	polymorphism	992262	992134	99.9726	-	-
15286		G	A	polymorphism	991591	991458	99.9801	-	-
15493		T	C	polymorphism	937664	937524	99.971	-	-
15647		G	A	polymorphism	984814	984711	99.9686	-	I-V
15836		G	A	polymorphism	992485	992348	99.9818	-	I-V
15912	TRNT	A	G	polymorphism	991144	990862	99.9446	-	-
16114	D-loop	G	A	polymorphism	526269	526179	99.9783	-	-
16125		C	T	polymorphism	430486	430445	99.9856	-	-
16228		C	T	polymorphism	591601	591408	99.9593	-	-
16250		G	A	polymorphism	589890	589772	99.9747	-	-
16303		T	A	polymorphism	19550	19507	99.7749	-	-

	16304		A	G	polymorphism	32169	31944	99.2757	-	-
	16347		T	C	polymorphism	999278	998936	99.9571	-	-
total numbers of alterations										
49										
TIG106	103	D-loop	A	G	polymorphism	816478	816159	99.9465	-	-
(80y)	146		T	C	polymorphism	463870	463707	99.9345	-	-
	199		C	T	polymorphism	102490	102396	99.8956	-	-
	204		C	T	polymorphism	149846	149724	99.9092	-	-
	260		G	A	polymorphism	275646	275569	99.9666	-	-
	488		T	C	polymorphism	579627	579544	99.966	-	-
	951	RNR1	C	CA	polymorphism	999712	942871	94.3143	1-ins	-
	1594		A	G	polymorphism	725287	725077	99.948	-	-
	4500	ND2	C	T	polymorphism	783641	783486	99.9678	-	-
	4828		A	G	polymorphism	774591	774212	99.9342	-	T-A
	5103		T	C	polymorphism	993185	992661	99.9177	-	-
	5596	TRNA	C	T	polymorphism	903630	902900	99.9106	-	-
	6570	COX1	A	AG	polymorphism	613820	567272	92.4167	1-ins	-
	6731		A	G	polymorphism	711979	711256	99.8812	-	-
	7304		T	C	polymorphism	944380	943966	99.9349	-	-
	7594	COX2	G	A	polymorphism	988360	987901	99.9402	-	-
	8569	ATP6	A	G	polymorphism	583487	583225	99.9366	-	-
	8686		A	G	polymorphism	794123	793979	99.9611	-	T-A
	8769		G	A	polymorphism	740110	740024	99.9793	-	-
	8814		T	C	polymorphism	707633	707557	99.9634	-	-
	9080		T	C	polymorphism	733666	733576	99.9568	-	L-P
	9150		T	C	polymorphism	766626	766545	99.9696	-	-
	9362	COX3	A	G	polymorphism	991057	990918	99.9764	-	-
	9560		G	A	polymorphism	992242	992109	99.9719	-	-
	9935		C	T	polymorphism	841928	841773	99.968	-	-
	10385	ND3	C	T	polymorphism	851737	851548	99.9668	-	-
	10858	ND4	T	C	polymorphism	807345	807186	99.9482	-	-
	11131		T	C	polymorphism	857807	857657	99.957	-	-
	11899		A	G	polymorphism	994752	993948	99.9054	-	-
	12177	TRNH	A	G	polymorphism	864733	864466	99.9503	-	-
	12296	TRNL2	T	C	polymorphism	791740	791453	99.9476	-	-
	12346	ND5	G	A	polymorphism	751729	751636	99.9721	-	-
	12690		C	T	polymorphism	875624	875431	99.9607	-	-
	13548		A	G	polymorphism	991754	991538	99.9636	-	-
	14185	ND6	T	C	polymorphism	742659	742519	99.9548	-	-
	14266		C	T	polymorphism	719984	719856	99.9649	-	-
	14455		C	T	polymorphism	673574	673428	99.9618	-	-
	14554		G	A	polymorphism	746687	746598	99.9708	-	-

	14751	CYTB	C	T	polymorphism	690636	690496	99.9625	-	T-I
	14768		T	C	polymorphism	662813	662751	99.9673	-	-
	15028		G	A	polymorphism	691085	690986	99.9731	-	-
	15208		T	C	polymorphism	723920	723801	99.9624	-	-
	15286		G	A	polymorphism	683504	683390	99.9706	-	-
	15493		T	C	polymorphism	654899	654802	99.9655	-	-
	15647		G	A	polymorphism	990701	990596	99.9807	-	I-V
	15836		G	A	polymorphism	994239	994128	99.9836	-	I-V
	15912	TRNT	A	G	polymorphism	984103	983775	99.9434	-	-
	16125	D-loop	C	T	polymorphism	480425	480357	99.969	-	-
	16228		C	T	polymorphism	4218	4203	99.6444	-	-
	16250		G	A	polymorphism	1041	1041	100	-	-
	16254		A	G	polymorphism	98	93	94.898	-	-
	16263		C	T	polymorphism	270	266	98.5185	-	-
	16303		T	A	polymorphism	4523	4490	99.2704	-	-
	16304		A	G	polymorphism	12169	11987	98.4304	-	-
	16347		T	C	polymorphism	984763	984452	99.9572	-	-
	16504		C	T	polymorphism	446389	446283	99.9689	-	-
total numbers of alterations										
56										
TIG107	103	D-loop	A	G	polymorphism	914198	913966	99.9638	-	-
(81y)	150		C	T	polymorphism	168807	168786	99.9727	-	-
	195		T	C	polymorphism	2624	2618	99.7713	-	-
	199		C	T	polymorphism	223	222	99.5516	-	-
	204		C	T	polymorphism	208	194	93.2692	-	-
	247		G	-	polymorphism	65715	65700	99.9772	1-del	-
	263		G	A	polymorphism	80714	80692	99.9678	-	-
	488		T	C	polymorphism	858798	858666	99.9773	-	-
	706	RNR1	A	G	polymorphism	991704	991388	99.9602	-	-
	951		C	CA	polymorphism	999422	916568	91.7098	1-ins	-
	1594		A	G	polymorphism	987856	987584	99.956	-	-
	1711	RNR2	C	T	polymorphism	987941	987740	99.9692	-	-
	2222		T	TA	polymorphism	988722	894706	90.4912	1-ins	-
	3547	ND1	T	A	polymorphism	733857	733825	99.9823	-	-
	4710	ND2	A	G	polymorphism	991538	991262	99.9618	-	-
	6021	COX1	G	A	polymorphism	987711	987564	99.9766	-	-
	6570		A	AG	polymorphism	919139	848946	92.3632	1-ins	-
	7190		C	A	polymorphism	990863	990820	99.9701	-	-
	7993	COX2	T	C	polymorphism	981318	981050	99.9608	-	-
	8686	ATP6	A	G	polymorphism	985240	985079	99.9678	-	T-A
	8769		G	A	polymorphism	990794	990680	99.9845	-	-
	8814		T	C	polymorphism	950785	950692	99.963	-	-

	9530	COX3	A	G	polymorphism	991323	990826	99.9137	-	-
	9935		C	T	polymorphism	988657	988517	99.9752	-	-
	10385	ND3	C	T	polymorphism	988787	988611	99.9745	-	-
	10858	ND4	T	C	polymorphism	914229	914020	99.9134	-	-
	11131		T	C	polymorphism	987286	987164	99.9259	-	-
	11954		G	A	polymorphism	992085	991088	99.8913	-	A-T
	12177	TRNH	A	G	polymorphism	988760	988429	99.9533	-	-
	12346	ND5	G	A	polymorphism	992565	992423	99.9796	-	-
	12657		A	G	polymorphism	991487	991190	99.954	-	-
	12690		C	T	polymorphism	990382	990220	99.9739	-	-
	13248		A	G	polymorphism	989211	989027	99.971	-	-
	14303	ND6	T	C	polymorphism	915461	915337	99.9737	-	N-S
	14455		C	T	polymorphism	896809	896639	99.9671	-	-
	14751	CYTB	C	T	polymorphism	874050	873895	99.9753	-	T-I
	14768		T	C	polymorphism	850599	850521	99.9757	-	-
	15028		G	A	polymorphism	948466	948339	99.9782	-	-
	15189		T	C	polymorphism	898147	897997	99.9747	-	I-T
	15208		T	C	polymorphism	902067	901954	99.9731	-	-
	15286		G	A	polymorphism	880085	879977	99.9788	-	-
	15472		A	T	polymorphism	924107	923997	99.96	-	-
	15493		T	C	polymorphism	875167	875068	99.9771	-	-
	15647		G	A	polymorphism	987082	986975	99.9823	-	I-V
	15836		G	A	polymorphism	991114	991012	99.9841	-	I-V
	15912	TRNT	A	G	polymorphism	975845	975526	99.9065	-	-
	15953	TRNP	T	C	polymorphism	990124	989981	99.9755	-	-
	16114	D-loop	G	A	polymorphism	215950	215786	99.9162	-	-
	16125		C	T	polymorphism	76515	76446	99.8994	-	-
	16198		G	A	polymorphism	5562	5551	99.7663	-	-
	16228		C	T	polymorphism	12130	12083	99.596	-	-
	16250		G	A	polymorphism	10896	10878	99.8348	-	-
	16283		T	C	polymorphism	3326	3322	99.8797	-	-
	16304		A	G	polymorphism	14835	14771	99.4742	-	-
	16312		C	T	polymorphism	176163	176073	99.9319	-	-
total numbers of alterations										
55										
TIG101	103	D-loop	A	G	polymorphism	283052	282960	99.9537	-	-
(86y)	152		T	C	polymorphism	170256	170206	99.8485	-	-
	199		C	T	polymorphism	48695	48644	99.8747	-	-
	204		C	T	polymorphism	66811	66778	99.9431	-	-
	488		T	C	polymorphism	202962	202930	99.9665	-	-
	706	RNR1	A	G	polymorphism	942654	942270	99.9392	-	-
	951		C	CA	polymorphism	507197	469032	92.4753	1-ins	-

1594		A	G	polymorphism	253737	253625	99.9295	-	-
3006	RNR2	G	A	polymorphism	451849	451787	99.9692	-	-
3201		C	T	polymorphism	445020	444866	99.9333	-	-
3526	ND1	G	A	polymorphism	136938	136924	99.9744	-	-
5173	ND2	C	A	polymorphism	638063	637929	99.969	-	L-M
6570	COX1	A	AG	polymorphism	307098	283273	92.2419	1-ins	-
8281	TRNK	A	G	polymorphism	168002	167866	99.4113	-	-
8399	ATP8	C	T	polymorphism	216663	216632	99.9557	-	L-F
8458		T	C	polymorphism	199936	199640	99.7324	-	-
8569	ATP6	A	G	polymorphism	167968	167884	99.9226	-	-
8686		A	G	polymorphism	321132	321055	99.9505	-	T-A
8769		G	A	polymorphism	259261	259222	99.9745	-	-
8814		T	C	polymorphism	247938	247900	99.954	-	-
9935	COX3	C	T	polymorphism	266683	266632	99.9599	-	-
9990	TRNG	A	G	polymorphism	218407	218372	99.9593	-	-
10385	ND3	C	T	polymorphism	265101	265049	99.9562	-	-
10858	ND4	T	C	polymorphism	274332	274276	99.91	-	-
11131		T	C	polymorphism	262648	262551	99.9014	-	-
11899		A	G	polymorphism	453560	453151	99.8948	-	-
12177	TRNH	A	G	polymorphism	679774	679568	99.9498	-	-
12346	ND5	G	A	polymorphism	242943	242909	99.9621	-	-
12690		C	T	polymorphism	328597	328510	99.941	-	-
12942		T	C	polymorphism	261013	260959	99.9559	-	-
14455	ND6	C	T	polymorphism	248949	248912	99.9671	-	-
14653		C	T	polymorphism	291080	291035	99.956	-	-
14751	CYTB	C	T	polymorphism	234196	234140	99.9445	-	T-I
14768		T	C	polymorphism	227114	227090	99.9555	-	-
14964		T	C	polymorphism	185169	185148	99.9703	-	-
15028		G	A	polymorphism	230819	230782	99.9536	-	-
15208		T	C	polymorphism	247968	247927	99.9593	-	-
15286		G	A	polymorphism	243412	243362	99.9622	-	-
15493		T	C	polymorphism	209571	209545	99.9709	-	-
15647		G	A	polymorphism	904077	903996	99.9857	-	I-V
15836		G	A	polymorphism	799137	799052	99.9687	-	I-V
15912	TRNT	A	G	polymorphism	798799	798504	99.8937	-	-
16114	D-loop	G	A	polymorphism	334343	334241	99.9557	-	-
16125		C	T	polymorphism	250113	250084	99.946	-	-
16228		C	T	polymorphism	179904	179855	99.9628	-	-
16250		G	A	polymorphism	179619	179592	99.9755	-	-
16303		T	A	polymorphism	7719	7699	99.715	-	-
16304		A	G	polymorphism	11603	11533	99.2933	-	-
16347		T	C	polymorphism	279298	279210	99.9549	-	-

total numbers of alterations

49

TIG102	93	D-loop	A	G	polymorphism	870939	870779	99.9623	-	-
(97y)	103		A	G	polymorphism	929201	928888	99.9498	-	-
	199		C	T	polymorphism	105068	104972	99.8515	-	-
	204		C	T	polymorphism	108160	108019	99.8567	-	-
	210		A	G	polymorphism	119267	109256	91.535	-	-
	951	RNR1	C	CA	polymorphism	999572	940299	94.0702	1-ins	-
	1594		A	G	polymorphism	890317	890072	99.9513	-	-
	3532	ND1	A	G	polymorphism	479494	479349	99.9531	-	-
	6570	COX1	A	AG	polymorphism	707285	653475	92.392	1-ins	-
	7540	TRND	T	C	polymorphism	988861	988761	99.9637	-	-
	7759	COX2	A	G	polymorphism	839921	839820	99.9646	-	-
	8769	ATP6	G	A	polymorphism	836492	836382	99.9756	-	-
	8814		T	C	polymorphism	789420	789329	99.9683	-	-
	9525	COX3	C	T	polymorphism	905941	905772	99.9673	-	-
	9947		G	A	polymorphism	925975	925892	99.9732	-	-
	11131	ND4	T	C	polymorphism	43669	43526	99.5008	-	-
	11134		G	A	polymorphism	41513	41367	99.5688	-	-
	11136		C	T	polymorphism	38692	38526	99.5296	-	-
	11899		A	G	polymorphism	994266	993519	99.9148	-	-
	12177	TRNH	A	G	polymorphism	942103	941832	99.9532	-	-
	12219	TRNS2	A	G	polymorphism	963701	895981	92.9587	-	-
	12346	ND5	G	A	polymorphism	872463	872339	99.9713	-	-
	14134	ND6	C	T	polymorphism	805200	805111	99.9769	-	-
	14143		C	T	polymorphism	804561	804411	99.9657	-	-
	14455		C	T	polymorphism	745795	745653	99.9635	-	-
	14653		C	T	polymorphism	832364	832251	99.9686	-	-
	14751	CYTB	C	T	polymorphism	795565	795374	99.9608	-	T-I
	15208		T	C	polymorphism	798289	798161	99.9648	-	-
	15220		A	G	polymorphism	778614	778488	99.9619	-	-
	15493		T	C	polymorphism	745753	745622	99.9611	-	-
	15647		G	A	polymorphism	988702	988595	99.9777	-	I-V
	15836		G	A	polymorphism	993822	993703	99.9792	-	I-V
	15912	TRNT	A	G	polymorphism	981449	981076	99.9355	-	-
	16114	D-loop	G	A	polymorphism	563847	563768	99.9796	-	-
	16208		T	C	polymorphism	1806	1804	99.8893	-	-
	16228		C	T	polymorphism	1052	1047	99.4297	-	-
	16241		C	T	polymorphism	3	3	100	-	-
	16250		G	A	polymorphism	737	737	100	-	-
	16251		C	A	polymorphism	846	795	93.9716	-	-
	16303		T	A	polymorphism	800543	800403	99.9653	-	-

16304	A	G	polymorphism	810834	810554	99.945	-	-
total numbers of alterations								
41								

[a]Registered under GenBank accession no. AB055387. [b]All of the mutations existing in more than 90% of mtDNA were polymorphic ones that have been reported in normal human subjects.

Table S2. Primers used for PCR and mtDNA sequencing.

Primer	Primer sequence (5'-3')	Primer binding	Fragment size (bp)
Primer-1 for	AAATGTTTAGACGGGCTCACATCA	610	2744
Primer-1 rev	CCATTGCGATTAGAATGGGTACAA	3353	
Primer-2 for	ACCTAACAAACCCACAGGTCCTAA	2756	2879
Primer-2 rev	TAAAGTGGCTGATTTGCGTTCAGT	5634	
Primer-3 for	AAAAACTAGCCCCCATCTCAATCA	4865	2840
Primer-3 rev	AGGGCATACAGGACTAGGAAGCAG	7704	
Primer-4 for	GTGGCCTGACTGGCATTGTATTAG	6949	2905
Primer-4 rev	CGGATGAAGCAGATAGTGAGGAAA	9853	
Primer-5 for	AGCCATGTGATTTCACTTCCACTC	9284	2941
Primer-5 rev	GGGCATGAGTTAGCAGTTCTTGTG	12224	
Primer-6 for	TCAGCCACATAGCCCTCGTAGTAA	11616	2501
Primer-6 rev	GGGTTAGGATGAGTGGGAAGAAGA	14116	
Primer-7 for	CTCCAAAGACCACATCATCGAAAC	13491	2937
Primer-7 rev	GTAGCACTCTTGTGCGGGATATTG	16427	
Primer-8 for	GCGTCCTTGCCCTATTACTATCCA	15600	2256
Primer-8 rev	TGCGCTTACTTTGTAGCCTTCATC	1301	

Table S3. Primers used for Real-time quantitative PCR.

Gene	Symbol	Primer sequence (5'-3')
aldehyde dehydrogenase 5 family, member A1	ALDH5A1	F AGACCATCCTGGCTAACACG
		R GGAGTCTCGCTCTGTCATCC
caprin family member 2	CAPRIN2	F CGAATGTGTTTCCCAGACCT
		R TTGGGAATAAGGTGCTCCAG
cytochrome c oxidase subunit VIIa polypeptide 1	COX7A1	F CGCTTTCAGAACCGAGTGC
		R CCCAGCCAAGGGAGTACAAG
enoyl-CoA, hydratase/3-hydroxyacyl CoA dehydrogenase	EHHADH	F CAGCTTCTCCCCAGACTCAC
		R TGAATTGGCTTGTTGCAGAG
glycine C-acetyltransferase	GCAT	F TCTCGCATGGCGGATGA
		R GCAGCGGTCAATGTCTTCCT
mitochondrial ribosomal protein L28	MRPL28	F ATGCCAACAACGACAAGCTCT
		R CTGTAGAACTCTCGCTCAAACAGC
serine hydroxymethyltransferase 2	SHMT2	F AGTCTATGCCCTATAAGCTCAACCC
		R GCCGGAAAAGTCGAGCAGT
ubiquitin C	UBC	F TGGAGCCGAGTGACACCATT
		R TCAGCGAGGGTACGACCATCTTC
